# Overview on the Development of Electrochemical Immunosensors by the Signal Amplification of Enzyme- or Nanozyme-Based Catalysis Plus Redox Cycling

**DOI:** 10.3390/molecules29122796

**Published:** 2024-06-12

**Authors:** Ning Xia, Fengli Gao, Jiwen Zhang, Jiaqiang Wang, Yaliang Huang

**Affiliations:** 1 College of Chemistry and Chemical Engineering, Anyang Normal University, Anyang 455000, China; 2 School of Pharmacy, Hunan University of Chinese Medicine, Changsha 410208, China

**Keywords:** electrochemical immunosensors, redox cycling, enzymes, nanozymes, signal amplification

## Abstract

Enzyme-linked electrochemical immunosensors have attracted considerable attention for the sensitive and selective detection of various targets in clinical diagnosis, food quality control, and environmental analysis. In order to improve the performances of conventional immunoassays, significant efforts have been made to couple enzyme-linked or nanozyme-based catalysis and redox cycling for signal amplification. The current review summarizes the recent advances in the development of enzyme- or nanozyme-based electrochemical immunosensors with redox cycling for signal amplification. The special features of redox cycling reactions and their synergistic functions in signal amplification are discussed. Additionally, the current challenges and future directions of enzyme- or nanozyme-based electrochemical immunosensors with redox cycling are addressed.

## 1. Introduction

Electrochemical immunosensors have attracted widespread interest in clinical diagnosis, food quality control, and environmental protection [1,2,3]. The desirable combination of specific antibody–antigen recognition with convenient electrochemical methods endows immunosensors with inherent advantages, such as excellent selectivity, operational simplicity, and inherent miniaturization [4]. Under an electrical transducer, the immune recognition event of antigen–antibody interaction can be translated into a detectable chemical or physical parameter to produce an electrical output signal. Nonetheless, the low sensitivity of traditional electrochemical immunoassays cannot meet the need of the ultrasensitive determination of trace analytes [5]. In order to fulfill the urgent requirement of immunosensors with a high sensitivity and low detection limit, various signal amplification strategies have been integrated with immunoassays in the past decades [6,7], including enzymatic catalysis [8], DNA-based amplification techniques [9,10], and functional nanomaterials [11,12,13,14,15,16]. Among them, the perfect integration of the high specificity of enzymatic catalysis with the high simplicity of electrochemical techniques has become a successful approach for designing novel immunosensors in disease diagnosis, medicine research, and environmental monitoring [17,18,19,20].

Because of the high turnover frequency, good reaction selectivity, and excellent substrate specificity, enzymes have been popularly used as catalytic labels to provide high, stable, and reproducible signals, such as horseradish peroxidase (HRP) [21,22], alkaline phosphatase (ALP) [23,24], and glucose oxidase (GOx) [25,26]. Antigens or antibodies can be conjugated with reporter enzymes for molecular recognition and signal readout. After the specific antigen–antibody interaction, the enzymatic products are determined at the final step of immunoassays. The function of enzyme labeling is to catalytically generate a multitude of signal units, which can be feasibly determined by different electrochemical techniques. The change in electrochemical signal exhibits a stoichiometric relationship to the target concentration. Therefore, the single reporter enzyme at one recognition event can generate numerous signal molecules, eventually amplifying the electrochemical signal [27]. For example, a single-enzyme HRP can promote the generation of 10^7^ signal molecules per minute [28]. Notwithstanding the signal amplification, the low signal intensity of enzymatic products will result in a low sensitivity of enzyme-linked electrochemical immunoassays. In this view, enzymatic reactions are always fused with other advanced amplification strategies to improve the sensitivity, such as nanomaterials [29], multi-enzymes [30], and redox cycling [31,32]. Among them, the strategy of coupling enzymatic catalysis with redox cycling has aroused widespread interest since it only requires the introduction of additional reagents to the electrolyte medium [33]. The redox cycle process involves the repetitive generation of electroactive substances through electrochemical, enzymatic, or chemical reactions. Consequently, a small amount of product from the enzymatic reaction can induce the generation of an enhanced electrochemical signal. For this consideration, electrochemical immunosensors, DNA sensors, and aptasensors have been widely developed through enzymatic reaction plus redox cycling [34,35,36,37]. The integration of redox cycling and enzymatic catalysis can also be introduced into optical bioassays, including fluorescence [38,39], colorimetry [40,41,42], and surface-enhanced Raman scattering spectroscopy [43].

Although many reviews have focused on the signal-amplified strategies, few of them have paid attention to the guiding and systematic summary of the advances in electrochemical immunosensors based on enzyme- or nanozyme-based catalysis plus redox cycling [18,25,44,45,46,47]. In this review, we systematically summarize the recent developments of electrochemical immunosensors by the signal amplification of enzyme- or nanozyme-based catalysis plus redox cycling. After a brief overview of the types of redox cycling reactions, the applications of enzymatic catalysis plus redox cycling in electrochemical immunosensors are discussed according to the functions of natural enzymes (e.g., oxidoreductases and hydrolases) and artificial nanozymes. Furthermore, this review addresses the future perspectives on the development of electrochemical immunosensors with catalytic reaction plus redox cycling.

## 2. Types of Redox Cycling Reactions

Enzymatic products (P) can be accumulated in solution for a prolonged time and then participate in redox cycling for signal amplification. In redox cycling, the repeatedly coupled oxidation and reduction of signal reporters can produce highly amplified electrochemical signals without changing the background. Based on the types of electrochemical reactions occurring near or on the electrode surface, the system can be divided into electro-oxidization and electro-reduction. In the redox cycling of substances produced by enzymatic catalysis, the oxidized (or reduced) species can be repetitively reduced (or oxidized) through enzymatic, chemical, or electrochemical methods. Before the summarization of electrochemical immunosensors based on enzymatic catalysis plus redox cycling, it is necessary to briefly describe several typical but important redox cycling reactions in enzyme-linked immunoassays according to the method of redox cycling of electroactive signaling species.

### 2.1. Electrochemical–Enzymatic Redox Cycling

Oxidoreductases containing Cu or Fe ions, such as laccase, tyrosinase, and HRP, can be used as electrocatalysts to catalyze the redox reactions of substrates [48]. In the presence of extra oxidants, small redox molecules can be used as mediators to transfer electrons between the active center of the enzyme and the sensing electrode, including ferrocene (Fc) and its derivatives, 3,3′,5,5′-tetramethylbenzidine (TMB), and hydroquinone (HQ) [49,50,51,52,53,54]. Most of the hydrophobic mediators can penetrate the modification layers to exchange electrons with the sensing electrode.

**Figure 1:**
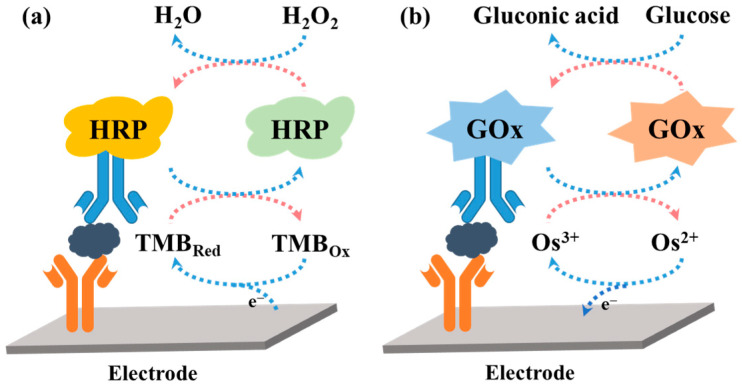
Schematic illustration of the EN redox cycling with HRP (**a**) and GOx (**b**) as the signal label of electrochemical biosensor.

The EN redox cycling schemes of oxidoreductase-based electrochemical biosensors are illustrated in Figure 1. In the HRP-based system, the mediator of TMB in the reduced form (TMB_Red_) is first enzymatically oxidized into TMB_Ox_ (the oxidized form of TMB) by HRP in the presence of H_2_O_2_. When TMB_Ox_ is electrochemically reduced back into its reducing format (TMB_Red_) at a suitable potential, the resulting TMB_Red_ is immediately oxidized again through HRP enzymatic catalysis. This process will cause a great increase in the reduction current. In the GOx-based system, the mediator Os^2+^ or Fc is first electrochemically oxidized into Os^3+^ or ferricinium ion. The oxidized mediator is then enzymatically reduced into its reduced form by GOx in the presence of glucose, leading to an increase in the oxidation current. The enzyme catalysis, herein named EN redox cycling of the mediator between enzymatic oxidization (or reduction) and electrochemical reduction (or oxidization), can produce an enhancement in the current at the electrode. However, in the presence of trace amounts of targets, few enzyme labels were immobilized on the electrode to participate in the enzymatic catalysis. In a limited time, the amount of the re-generated mediators is relatively low even in the presence of a nonlimiting concentration of enzyme substrates. Thus, the signal amplification efficiency of the EN redox cycling can be enhanced by improving the turnover number of enzyme labels or using nanomaterials as nanocarriers to increase the amount of enzyme labels per immunocomplex.

**Figure 2:**
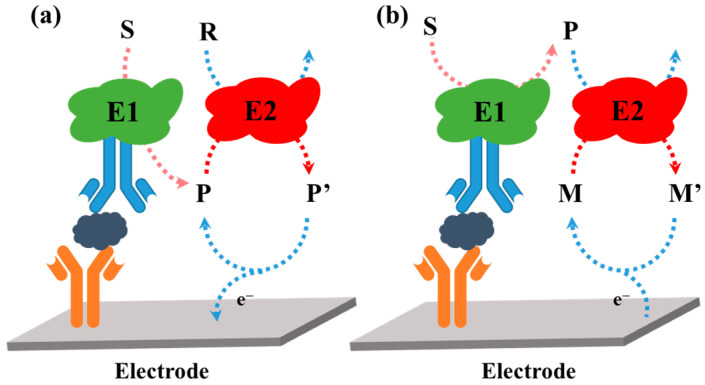
Schematic illustration of (**a**,**b**) two types of electrochemical–bienzymatic redox cycling systems.

Hydrolytic enzymes, such as ALP and *β*-Galactosidase (*β*-Gal), can be used as the primary enzyme reporter (E1) in combination with oxidoreductases to construct two enzymes-based strategies through the in situ detection of the hydrolytic products, which is named electrochemical–bienzymatic redox cycling [55,56]. In this method, oxidoreductases, such as GOx, glucose dehydrogenase (GDH), and diaphorase (DI), are required as the second enzyme (E2) for the one-step in situ regeneration of electroactive E1 product [57]. According to the function of enzymatic products, the strategies of the electrochemical–bienzymatic redox cycling system can be classified into two modes (Figure 2) [58]. In Figure 2a, the enzymatic product (P) generated from E1 serves as both the substrate of E2 and the electron mediator and can be recycled via repeated enzymatic reactions. In order to minimize the interference between the two enzymatic reactions, E1 should show no redox activity. Meanwhile, the optimal conditions for the two enzymes should be similar to ensure a high enzymatic efficiency. In Figure 2b, P serving as the co-substrate of E2 is continuously consumed during the enzymatic reactions. The bienzymatic redox cycling system in Figure 2a exhibits a higher amplification efficiency than that in Figure 2b.

### 2.2. Electrochemical–Chemical Redox Cycling

**Figure 3:**
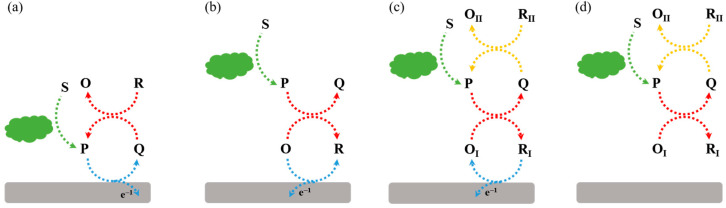
Schematic illustration of (**a**,**b**) two types of electrochemical–chemical redox cycling, (**c**) electrochemical–chemical-chemical redox cycling, and (**d**) chemical–chemical redox cycling.

Redox cycling of enzymatic products can be achieved through simple chemical reactions without the use of additional enzymes or electrodes. For example, the enzymatic product (P) can be first electrochemically oxidized into its oxidized form (Q). The additional reducing agent can chemically reduce Q into P. The regeneration of P during electrochemical scanning will induce a significant enhancement in the current, which is defined as electrochemical–chemical (EC) redox cycling amplification (Figure 3a). In another type of EC redox cycling system (Figure 3b) [59], the enzymatic product P serves as a chemical reducing agent to continuously regenerate the redox mediator (R) from its electrochemically oxidized product (O) near the electrode. In this process, the redox mediator serves as an electrocatalyst to transfer the electron from enzymatic product P to the electrode. The amplification efficiency of the second redox cycling is lower than that of the first one, and it is always used in the determination of enzyme activity rather than enzyme-linked electrochemical biosensors.

Chemical–chemical (CC) redox cycling can be achieved between two additional reagents. In contrast to EC redox cycling, electrochemical–chemical–chemical (ECC) redox cycling is more effective at amplifying the electrochemical signal (Figure 3c) [60,61,62,63]. Generally, the electroactive mediator (R_I_) is electrochemically oxidized into O_I_, which can be reduced immediately by the enzymatic product (P). The oxidized enzymatic product (Q) is then rapidly reduced back to P in the presence of excess reducing agent (R_II_). In this method, R_I_ serving as the redox mediator can be modified on the electrode surface (Figure 3c) or dispersed in the solution (Figure 3d). Redox cycling of the enzymatic product P between R_I_ and R_II_ can cause a significant increase in the anodic current. For the achievement of a low background signal and increased sensitivity, it is crucial to select an appropriate reducing agent, enzymatic substrate, redox mediator, and sensing electrode [64]. The reducing agent R_II_ should be electrochemically inactive in the scanning potential window and the chemical reaction of R_I_/P and Q/R_II_ should be very fast.

In addition, the enzymatic product P can promote the formation of electroactive metal deposition on the electrode surface to produce a detectable electrochemical signal. In this method, the enzymatic product P is regenerated from its oxidized form (Q) by the additional reducing substance R_II_. One of the most typical examples is enzymatic silver (Ag) biometallization, in which Ag^+^ (O_I_) is reduced by the enzymatic product P into metallic silver (R_I_) deposition on the solid substrate [65,66,67]. The electrochemical oxidation of the deposited Ag could produce a high electrochemical signal. In addition, such redox cycling systems have been widely used in optical bioassays, such as chemiluminescence [68,69,70,71,72], fluorescence [38,39], and SERS assays [43].

### 2.3. Electrochemical–Electrochemical Redox Cycling

**Figure 4:**
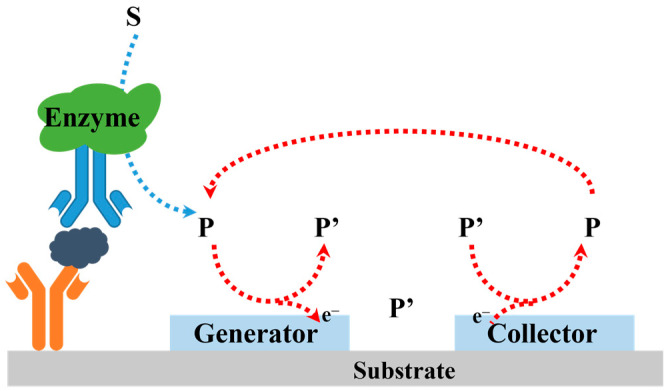
Schematic illustration of the EE-redo -cycling-based enzyme-linked electrochemical biosensor.

The electrochemical–electrochemical (EE) redox cycling of electroactive species produced by enzymatic catalysis is another approach for improving the sensitivity of electrochemical immunoassays [73,74,75]. In the EE redox cycling, two electrodes in close proximity to each other can serve as the generator and collector. The signaling species are electrochemically oxidized or reduced iteratively at the generator electrode, and are then mass-transported through diffusion onto the collector electrode to be electrochemically reduced or oxidized. Under the repeated redox reactions between the generator–collector electrodes, the electrochemical signal could be greatly amplified (Figure 4) [76,77]. The electrode gap should be as narrow as possible to facilitate the diffusion of the redox couple and enhance the redox cycling efficiency. To date, various electrode systems have been reported for redox cycling, including interdigitated array (IDA) electrodes [78], twin-electrode thin-layer cells [79], vertically paired electrodes [80], rotating ring-disc electrodes [81], and others [82,83].

## 3. Oxidoreductases as the Signal Labels of Electrochemical Immunosensors

### 3.1. HRP

**Figure 5:**
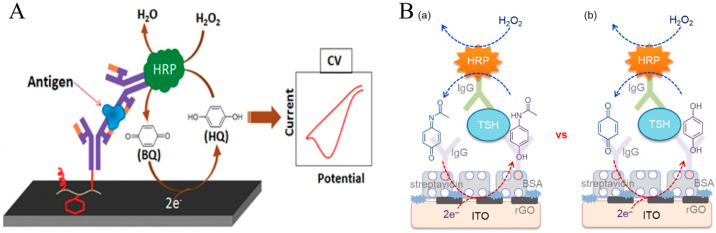
(**A**) Schematic illustration of the EN-redox-cycling-based electrochemical detection of mouse IgG using the ERGO-modified electrode [104]. Copyright 2016 American Chemical Society. (**B**) Schematic illustration of an EN-redox-cycling-based electrochemical immunoassay for TSH detection using (**a**) acetaminophen or (**b**) HQ as the HRP label substrate [106]. Copyright 2020 Elsevier.

HRP is one of the most commonly used enzyme labels in EN redox cycling for signal amplification [84]. The enzyme active center can be activated by the substrate (H_2_O_2_) to a ferryloxy form that can be electrochemically reduced back in a catalytic cycle by using a redox mediator or substrate to shuttle the electron from the electrode to the redox center of the enzyme [85,86]. The oxidized form of the mediator or substrate can be quantified by recording the redox current for the determination of the enzyme activity. A variety of redox molecules have been utilized as the mediators of HRP-based redox cycling, such as TMB [87,88], HQ [89,90,91,92], catechol [93,94,95], thionine [96,97], *o*-phenylenediamine (OPD) [98,99], 3-hydroxyl-2-aminopyridine [100], 5-methyl-phenazinium methyl sulfate [101], and *o*-aminophenol [102]. As a proof, Doldan et al. reported HRP-based EN redox cycling for the determination of exosomes with signal-on and signal-off formats [103]. In this work, CD9 proteins on the surface of exosomes were specifically labeled with HRP-conjugated α-mouse IgG antibodies. The HRP-based EN redox cycling of TMB in the presence of H_2_O_2_ resulted in a significantly amplified electrochemical signal. In addition, HQ is another commonly used mediator for designing EN-redox-cycling-based biosensors with HRP enzymatic catalysis. Haque et al. developed an electrochemical immunosensor for the detection of mouse IgG (Figure 5A) [104]. In this study, graphene oxide (GO) was deposited on the surface of an amine-terminated benzenediazonium-modified indium tin oxide (ITO) electrode via electrostatic and π-π stacking interactions. The deposited GO was then converted into electrochemically reduced graphene oxide (ERGO) through an electrochemical reduction method, followed by the modification of amphiphilic polymers for the covalent immobilization of antibodies. After the formation of the immunocomplex, HRP molecules were immobilized on the electrode surface. Under HRP-based EN redox cycling, the mediator HQ could be repetitively oxidized into *p*-benzoquinone (BQ) by HRP enzymatic catalysis. The resulting BQ was regenerated during the electrochemical reduction progress, thus producing an amplified current. However, in the HQ-mediated redox cycling system, the electrochemical reduction of H_2_O_2_ and the electrochemical oxidation of HQ may cause a high background and affect the analytical performance. Thus, a redox mediator with a formal potential lower than that of H_2_O_2_ is desirable for addressing this problem. Typically, Kang et al. used catechol as the mediator to develop an HRP-based EN redox cycling for the detection of mouse IgG [105]. Yan et al. reported an EN-redox-cycling-based electrochemical immunosensor for the determination of thyroid-stimulating hormone (TSH) with acetaminophen as the HRP substrate (Figure 5B) [106]. In this study, ERGO was used to partially decorate the ITO electrode for maintaining a high electrocatalytic activity for BQ reduction even at the immunosensing-layer-modified electrode. As an *N*-acetylated derivative of *p*-AP, acetaminophen with better stability against light exhibited a formal potential of 0.28 V, which is higher than that of HQ (0.03 V). The redox reaction of acetaminophen is highly reversible at neutral pH. The applied potential was set at 0 V to minimize the electrochemical reduction of H_2_O_2_ and avoid the electrochemical oxidation of acetaminophen. In HRP-based EN redox cycling, both the enzymatic oxidation of acetaminophen by HRP in the presence of H_2_O_2_ and the electrochemical reduction of the catalytic product were very fast.

**Figure 6:**
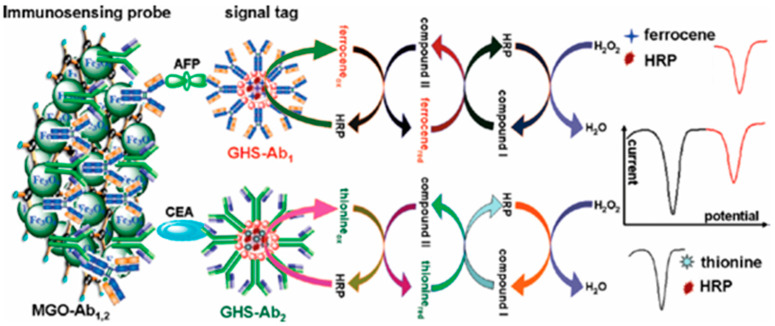
Schematic illustration of the multiplexed electrochemical immunoassay protocol and the measurement principle of the sandwich immunoassay [116]. Copyright 2011 American Chemical Society.

The above redox cycling strategies always show a modest signal amplification efficiency because of the relatively low enzyme/antibody ratio (1:1). To increase the number of HRP molecules in the signal output step, nanomaterials with a large surface area have been used as carriers to load enzymes and detection antibodies for a wide detection range and high sensitivity, such as gold nanomaterials [107,108], magnetic nanoparticles [109], carbon nanotubes [110], graphene [111,112], silica nanoparticles [113,114], and so forth [115]. Tang et al. reported a magneto-controlled flow-through multiplexed immunoassay method for the simultaneous determination of carcinoembryonic antigen (CEA) and alpha-fetoprotein (AFP) using magnetic graphene nanosheets as capture probes and multifunctional nanogold hollow microspheres as distinguishable signal labels (Figure 6) [116]. In this work, nanogold hollow microspheres were used to load HRP, two electroactive molecules (Fc and thionine), and CEA/AFP antibodies. Antibody-modified magnetic graphene nanosheets served as filter-like networks to capture AFP and CEA. Thionine/Fc-mediated HRP-based redox cycling in the presence of H_2_O_2_ endowed the immunoassays with wide working ranges and low detection limits for the simultaneous detection of AFP and CEA.

**Figure 7:**
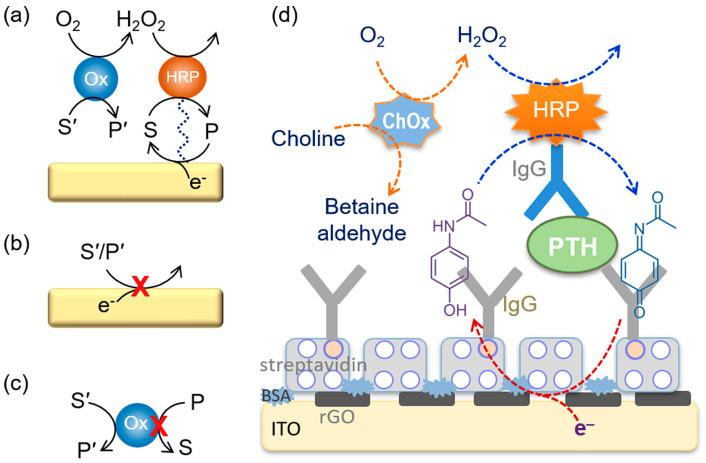
Schematic illustration of (**a**) the reaction in which a preceding enzyme (Ox) catalyzes the corresponding substrate (S′) to the oxidized product (P′), during which H_2_O_2_ is generated and oxidizes the peroxidase substrate (S) to the electroactive signaling product (P) in the presence of HRP, (**b**) the electrochemical reaction of S′ and P′ on an electrode, (**c**) Ox-catalyzed reduction of P to S in the presence of S′, and (**d**) an electrochemical immunoassay for PTH detection using the ChOx-HRP cascade reaction and acetaminophen as the HRP substrate [117]. Copyright 2020 Elsevier.

In HRP-based immunosensors, the enzymatic products can be electrochemically reduced within the applied potential window. However, most of the mediators are electroactive, and H_2_O_2_ is easily reduced within the electrochemical potential window, resulting in high background current. Meanwhile, the electro-reduction of O_2_ dissolved in the solution may increase the background current when a highly electrocatalytic electrode is employed as the working electrode. To resolve this problem, several enzymes that can in situ catalyze the formation of H_2_O_2_ were integrated with the HRP-based immunoassays based on a bienzymatic cascade strategy. In order to avoid the potential side reactions in this method, it is critical to select the appropriate HRP substrate and preceding oxidase. GOx has been widely used as a preceding oxidase to catalyze the oxidation of the corresponding substrate in the presence of O_2_, which was accompanied with the production of H_2_O_2_ for the next HRP enzymatic catalysis [117,118,119,120]. However, the GOx-catalyzed reduction of the oxidized peroxidase substrate may hamper the immunosensing performance. Alternatively, Yan et al. reported an electrochemical immunosensor for the detection of parathyroid hormone (PTH) based on a choline oxidase (ChOx)-HRP bienzymatic cascade (Figure 7) [117]. In this work, ChOx catalyzed the oxidation of choline, and the in situ generated H_2_O_2_ could subsequently oxidize acetaminophen through the HRP enzymatic catalysis. The performances between the ChOx-HRP and GOx-HRP systems were compared. It was demonstrated that the oxidized acetaminophen could not be reduced by ChOx in the presence of choline and that the signal-to-background ratio for the ChOx-HRP system was higher than that for the GOx-HRP system using acetaminophen as the HRP substrate.

### 3.2. GOx

GOx can catalyze the oxidation of glucose with O_2_ or other species as the electron acceptor [121]. The enzyme shows excellent stability and high catalytic activity over a broad pH range (pH 4~7) and thus has been widely used in immunoassays [122,123]. In the first-generation glucose monitoring system, GOx enzymatic catalysis was monitored by determining the level of the co-substrate (O_2_) or by-product (H_2_O_2_). However, other reductants in biological liquids can also be oxidized on the electrode at a similar potential, leading to a false positive signal. Meanwhile, differences in the oxygen tension of samples may bring fluctuations into the electrode response. As the substitute of O_2_, other redox mediators, such as Fc, osmium complexes, and Ru(NH_3_)_6_^3+^, have been used to design GOx-based electrochemical biosensors by accelerating the electrical communication between the electrode and the catalytic center of GOx [124,125,126,127].

**Figure 8:**
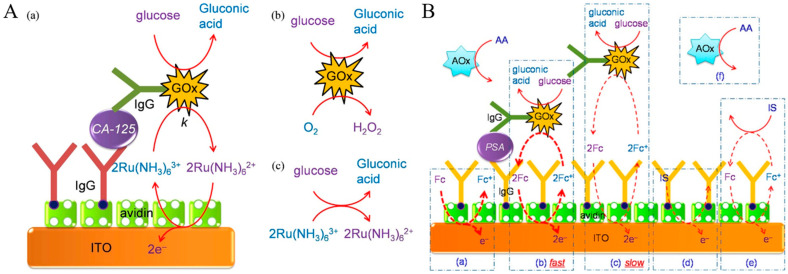
(**A**) Schematic illustration of (**a**) an electrochemical immunosensor using GOx label-based EN redox cycling, (**b**) enzymatic glucose oxidation by O_2_, (**c**) homogeneous reaction of glucose and Ru(NH_3_)_6_^3+^ [128]. Copyright 2013 American Chemical Society. (**B**) Schematic illustration of a washing-free immunosensor using proximity-dependent electron mediation and the reactions involved (**a**–**f**) [129]. Copyright 2014 American Chemical Society.

A relatively long incubation time for enzymatic catalysis can favor the generation of an increasing number of signal species, which is unfavorable in time-saving detection applications. To address this shortcoming, Singh et al. developed an incubation-period-free electrochemical immunosensor for the detection of cancer antigen 125 (CA-125) based on GOx-based EN redox cycling, in which glucose was used as the reducing substrate and Ru(NH_3_)_6_^3+^ was used as the redox mediator [128]. As shown in Figure 8A, ITO with a low and reproducible capacitive background current/charge was utilized as the sensing electrode. The applied potential was set at 0.05 V for the chronocoulometric measurement, which is higher than the formal potential of Ru(NH_3_)_6_^3+^/Ru(NH_3_)_6_^2+^ (−0.15 V). The redox couple undergoes a fast outer-sphere electron transfer reaction at the ITO electrode. The enzymatic reduction of Ru(NH_3_)_6_^3+^ in air-saturated buffer was faster than that of the enzymatic reduction of O_2_. Meanwhile, the direct electro-oxidation of glucose at the ITO electrode surface and the direct reaction between glucose and Ru(NH_3_)_6_^3+^ are slow, achieving a low background signal. After the attachment of GOx-modified IgG on the electrode, Ru(NH_3_)_6_^3+^ was reduced to Ru(NH_3_)_6_^2+^ with the transformation of glucose into gluconic acid. Then, Ru(NH_3_)_6_^2+^ was re-oxidized back to Ru(NH_3_)_6_^3+^ at the electrode surface. The repeated EN redox cycling produced a high chronocoulometric charge. Finally, the rapid Ru(NH_3_)_6_^2+^-mediated electron transfer between the electrode and the GOx label and the acquisition of chronocoulometric charge at a potential in the mass transfer-controlled region obviously minimized the incubation period and improved the signal-to-background ratio.

In heterogeneous ELISA, multiple washing steps are required to remove the unbound labels and interfering species. Accordingly, Yang’s group reported a washing-free immunosensor for the sensitive and single-step detection of prostate-specific antigen (PSA) in serum based on the EN redox cycling and proximity-dependent electron mediation between GOx and ITO electrodes [129]. As shown in Figure 8B, the captured GOx reporter has a faster electron mediation with the electrode than the unbound GOx because of the distance-dependent electron mediation of ferrocenemethanol (FcM) between GOx and ITO electrodes. The L-ascorbate oxidase (AOx)-catalyzed oxidation of L-ascorbic acid (AA) minimized the influence of AA. This washing-free immunosensor based on the EN redox cycling could sensitively determine PSA after an incubation period of 10 min.

### 3.3. Tyrosinase

**Figure 9:**
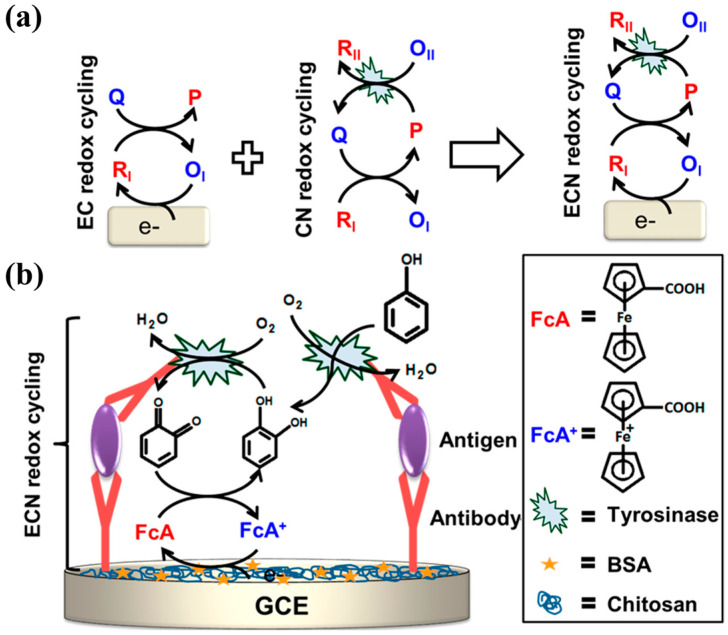
Schematic illustration of (**a**) the integration of EC and CN redox cycling containing mediator R_I_ and its oxidized form O_I_, oxidant Q and its reduced form P, and oxidant of enzyme O_II_ and its reduced form R_II_; and (**b**) example of the integrated ECN redox cycling for electrochemical enzymatic signal enhancement in the immunosensing of protein [138]. Copyright 2017 American Chemical Society.

As a copper-containing redox enzyme, tyrosinase manifests two catalytic properties (monooxygenase and oxidase activity), and has been widely used to construct enzyme electrodes for the determination of catechol and phenol via a redox cycling process [130,131,132,133,134,135]. The high and selective catalytic ability led to the application of tyrosinase in the affinity assay as a catalytic label or signal amplifier [136]. Tyrosinase can be used to design EN redox cycling schemes, in which tyrosinase serves simultaneously as the label to enzymatically generate the electroactive product (catechol) and to regenerate the oxidized form of the tyrosinase product (*ortho*-quinone). The regenerated *ortho*-quinone can be electrochemically reduced to produce an amplified electrochemical response. For instance, Chumyim developed an electrochemical immunosensor for the detection of *Salmonella* Typhimurium cells based on tyrosinase multilayer-functionalized CNTs as electrochemical labels and EN redox cycling [137]. Akanda et al. reported integrated electrochemical–chemical–enzymatic (ECN) redox cycling for protein detection [138]. As illustrated in Figure 9, tyrosinase catalyzed the oxidation of phenol to *o*-benzoquinone in the presence of O_2_. Fc was used as the redox mediator to catalyze the reduction of *o*-benzoquinone through electro-reduction-based EC redox cycling. The combination of non-enzymatic EC redox cycling with the enzymatic CN redox system significantly amplified the signal and improved the biosensing performance.

**Figure 10:**
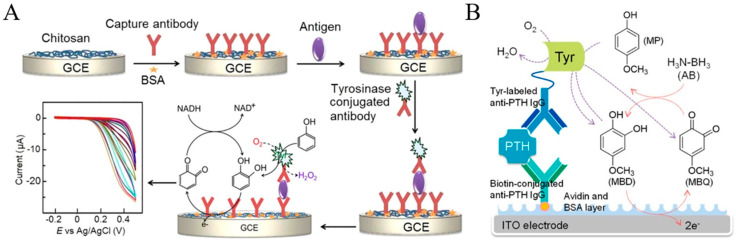
(**A**) Schematic illustration of the preparation of the electrochemical immunosensor and immunoassay procedure with the tyrosinase label and EC redox cycling [139]. Copyright 2016 American Chemical Society. (**B**) Schematic illustration of an electrochemical immunosensor using Tyr (tyrosinase) as a catalytic label and 4-methoxyphenol as a reducing agent. Reprinted with permission from reference [140]. Copyright 2016 American Chemical Society.

Akanda et al. reported tyrosinase-responsive electrochemical oxidation-based EC redox cycling for the detection of CEA (Figure 10A) [139]. In this study, phenol and nicotinamide adenine dinucleotide disodium salt in the reduced format (NADH) were employed as the enzyme substrate and the reducing agent, respectively. The low electroactivity of phenol and the high oxidation over-potential of NADH on the chitosan-modified GCE resulted in a negligible background. Tyrosinase with monooxygenase activity could catalyze the conversion of the poorly electroactive phenol into the highly electroactive product catechol at neutral pH. The EC redox cycling of catechol by NADH led to a greatly amplified voltammetric signal and high signal-to-noise ratio. The unfavorable tyrosinase-catalytic oxidation of catechol can be reduced back to catechol in the presence of excess NADH. Finally, the developed method was capable of determining CEA in a linear range of 1.0 pg/mL~0.1 μg/mL with a detection limit of 100 fg/mL. To further investigate the detailed information of tyrosinase as a catalytic label in immunoassay, Park et al. compared the applicability of four *para*-substituted phenolic compounds as tyrosinase substrates and three reducing agents for EC redox cycling (Figure 10B) [140]. In this work, 4-methoxyphenol and ammonia-borane (H_3_N−BH_3_, AB) were selected as the tyrosinase substrate and the reducing agent. The rapid EC redox cycling of the tyrosinase product led to a high electrochemical signal level. Meanwhile, the slow oxidation of AB on the low electrocatalytic ITO electrode at a low applied potential resulted in a low background. As a result, PTH was determined in a linear range of 2 pg/mL–1 μg/mL with a detection limit of 2 pg/mL.

### 3.4. GDH

The above oxidoreductases, including HRP, GOx, and tyrosinase, exhibit the highest activity when they are expressed and folded into the proper three-dimensional structure. In addition, many inactive enzymes (apoenzymes) require the covalent or non-covalent coupling of non-diffusional cofactors to trigger their catalytic activity. The possibility to reversibly modulate the activity of enzymes has been proven to be a valuable strategy for optical and electrochemical biosensors [141,142,143]. Typically, GDH is one of the prominent examples of apoenzymes. According to the redox cofactors, GDH can be subdivided into flavin adenine dinucleotide (FAD)-dependent GDH (FAD-GDH), pyrroloquinoline quinone (PQQ)-dependent GDH (PQQ-GDH), and nicotine adenine dinucleotide (NAD) or nicotine adenine dinucleotide phosphate (NADP)-dependent GDH [144]. In contrast to GOx, GDH is insensitive to O_2_ and exhibits a higher redox potential and catalytic activity [145]. Moreover, the reduced form of GDH cannot be oxidized by the dissolved O_2_. Thus, GDH has been widely used to replace GOx for the design of electrochemical immunosensors [146]. The catalytic activity of PQQ-GDH can be activated through its reconstitution [147]. After the activation of apo-GDH by PQQ and Ca^2+^ ions, PQQ-GDH can catalyze the oxidation of glucose with a particularly high catalytic efficiency and turnover number. It can be regenerated into its oxidized form by a series of electron acceptors or mediators. The detailed mechanism and application of PQQ-GDH in redox-mediated electrochemical reactions have been reported by Limoges’s group [148,149].

**Figure 11:**
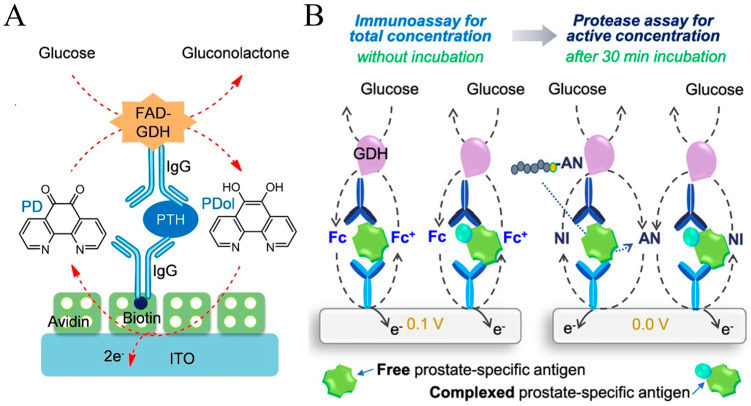
(**A**) Schematic illustration of an EN redox cycling-based electrochemical immunosensor [150]. Copyright 2021 Wiley-VCH. (**B**) Schematic illustration of the interference-free duplex detection method using (left) a sandwich-type immunoassay for total PSA and (right) an enzymatic-reaction-based protease assay for free PSA [151]. Copyright 2021 American Chemical Society.

Compared with other GDHs, the catalysis of FAD-GDH does not require external cofactors. Haque et al. developed an electrochemical immunosensor for the detection of PTH with FAD-GDH-based EN redox cycling (Figure 11A) [150]. In this study, 1,10-phenanthroline-5,6-dione (PD), a heterocyclic electroactive quinone, was used as the electron mediator and ITO with a low electrocatalytic activity was employed as the working electrode to decrease the background current from the reduction of O_2_. In the presence of glucose, FAD-GDH catalyzed the rapid reduction of PD to 1,10-phenanthroline-5,6-diol (PDol). In this process, PD was not reduced by glucose. Compared with other quinone-based electron mediators, PD-based EN redox cycling showed the highest signal-to-background ratio. In addition, Park et al. reported the interference-free duplex detection of total and active enzymes at a working electrode based on two different EN redox cycling reactions [151]. As illustrated in Figure 11B, the GDH label on the immunocomplex could initiate the EN redox cycling reaction in the presence of glucose and FcM, providing a high electrochemical signal without an incubation period at a higher applied potential (0.1 V vs. Ag/AgCl). Then, free PSA with proteolytic activity promoted the hydrolysis of the electro-inactive peptide substrates, resulting in the release of electroactive segments over an incubation period of 30 min. Under the EN redox cycling reaction in the presence of GDH, glucose, and 4-amino-1-naphthol (4-NH_2_-1-N), a strong electrochemical signal was obtained at a low applied potential (0.0 V).

### 3.5. FAD-Dependent Glycerol-3-Phosphate Dehydrogenase (GPDH)

**Figure 12:**
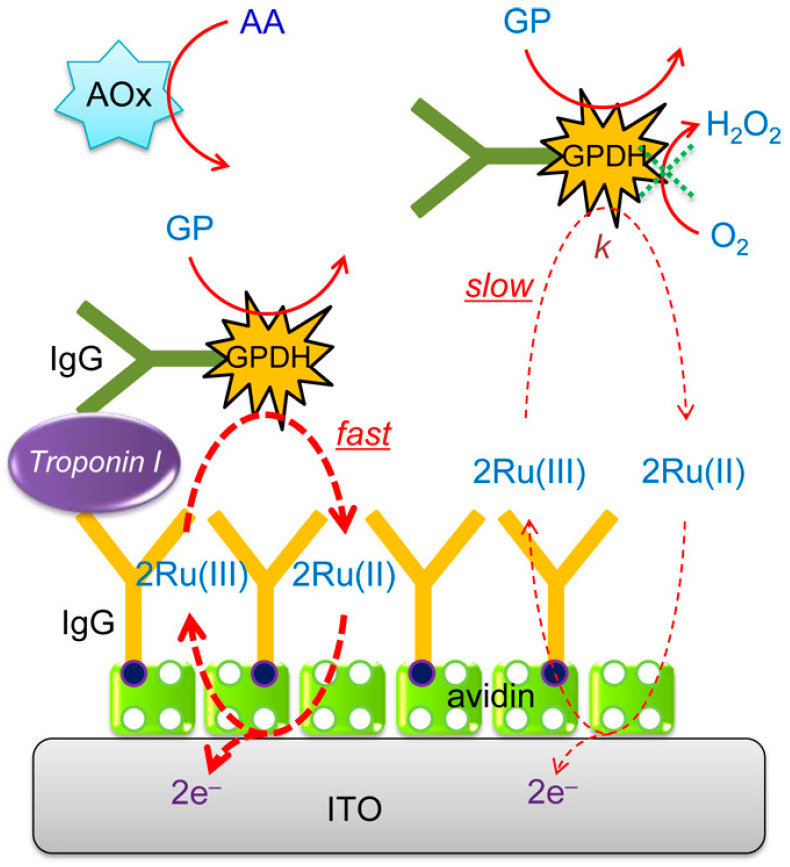
Schematic illustration of a washing-free electrochemical immunosensor based on EN redox cycling in the presence of GP, GPDH, and Ru(NH_3_)_6_^3+^ [153]. Copyright 2015 American Chemical Society.

The proximity-dependent electron mediation of FcM between the electrode and the enzyme label can facilitate the differentiation between the bound and unbound labels without washing steps. Based on this concept, Dutta et al. developed a washing-free heterogeneous immunosensor for the detection of PSA [129]. However, the high concentration of the enzyme substrate (glucose) was used to avoid the influence of pre-existing glucose in real physiological samples on the mediated oxidation of glucose by GOx. In addition, O_2_ dissolved in solution could competitively participate in the GOx-catalyzed oxidation of glucose, leading to a low sensitivity and poor reproducibility. Moreover, the applied potential of 0.13 V may cause the electro-oxidation of other interfering species. It has been reported that the reaction between FAD-GPDH and dissolved O_2_ is slow and the level of glycerol-3-phosphate (GP) in blood is low [152]. To avoid the interference of dissolved O_2_ and metabolites, Dutta et al. developed a low-interference washing-free electrochemical immunosensor for the detection of cardiac troponin I using FAD-GPDH, GP, and Ru(NH_3_)_6_^3+^ as the signal label, an enzyme substrate, and an electron mediator (Figure 12) [153]. Under the catalysis of FAD-GPDH in the presence of GP, Ru(NH_3_)_6_^3+^ was converted into Ru(NH_3_)_6_^3+^, whose concentration near the electrode was higher than that in solution. The EN redox cycling of Ru(NH_3_)_6_^3+^ allowed for continuous electron mediation. Therefore, the mediation between the ITO electrode and the bound GPDH was fast and that for the unbound antibody was slow. This method avoided the oxidation of uric acid and acetaminophen by using an applied potential near 0 V. In addition, AOx was added to oxidize AA and eliminate its interference. Under the optimized conditions, cardiac troponin I was determined in a linear range of 0.01–100 ng/mL.

### 3.6. DT-Diaphorase (DT-D)

**Figure 13:**
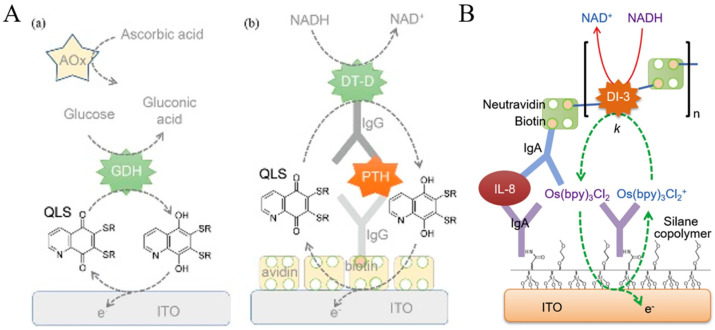
(**A**) Schematic illustration of an electrochemical glucose sensor using GDH and QLS (**a**), and (**b**) that of an electrochemical immunosensor for PTH detection using DT-D and QLS [156]. Copyright 2022 Wiley. (**B**) Schematic illustration of a sandwich-type immunoassay for IL-8 detection using a polyenzyme label based on diaphorase and neutravidin [157]. Copyright 2021 Elsevier.

DT-D is a flavin-containing oxidoreductase that can catalyze the reduction of redox mediators (e.g., metal complexes, quinones, and nitro(so) compounds) in the presence of NADH or NADPH [154]. Because of its unique properties, DT-D has been used in EN redox cycling for electrochemical immunosensors. For instance, Ichzan et al. designed an EN redox cycling system involving ITO electrodes, 1,4-naphthoquinone, DT-D, and NADH [155]. Nandhakumar et al. developed an electrochemical immunosensor with di(thioether sulfonate)-substituted quinoline-1,4-dione (QLS) as the electron mediator for DT-D-involving EN redox cycling [156]. As shown in Figure 13A, the naphthoquinone core was substituted with a thioether sulfonate group for the achievement of high hydrophilicity, rapid dissolubility, high stability, moderate formal potential, and high electron mediation ability. Then, QLS was used as the electron mediator for constructing GDH-based electrochemical glucose biosensors and DT-D-based electrochemical immunosensors. Under EN redox cycling in the presence of DT-D and NADH, the repetitive generation of the reduced form of QLS resulted in an amplified oxidation current. This method was capable of determining PTH with a detection limit of 2 pg/mL, which was lower than the normal PTH concentration in humans. The bimolecular rate constants between DT-D and some metal complexes as the electron acceptors are high (up to 10^9^ M^−1^s^−1^), which is favorable in electrochemical immunosensors based on EC redox cycling. In addition, Bhatia et al. reported an electrochemical immunosensor for interleukin-8 (IL-8) detection using a DT-D-based polyenzyme label and EN redox cycling (Figure 13B) [157]. In this work, biotinylated DT-D and neutravidin were used to produce the polyenzyme labels. After the electrochemical oxidation of Os(bpy)_2_Cl_2_, the EN redox cycling of Os(bpy)_2_Cl_2_ in the presence of DT-D and NADH led to signal amplification. Under the optimized conditions, IL-8 was sensitively detected in a wide linear range from 1 pg/mL to 1 μg/mL with a detection limit of 1 pg/mL.

**Figure 14:**
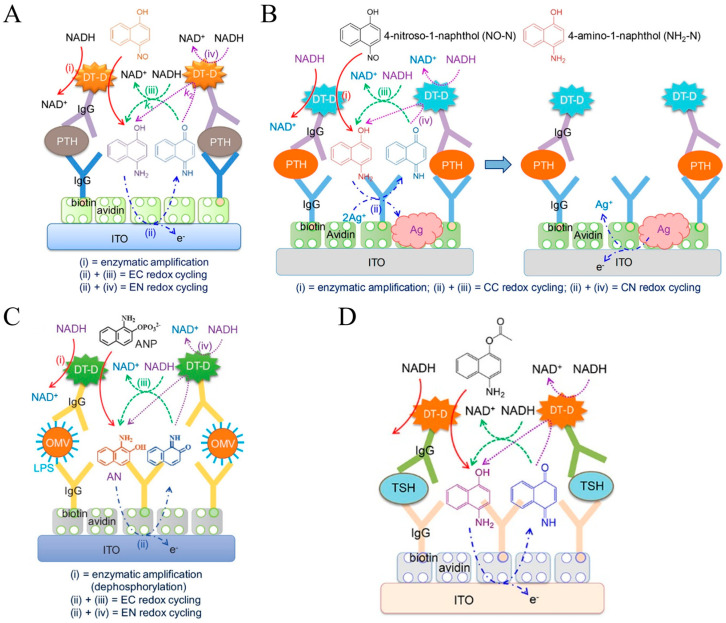
(**A**) Schematic illustration of an electrochemical immunosensor using DT-D as an enzyme label [159]. Copyright 2017 American Chemical Society. (**B**) Schematic illustration of a sandwich-type electrochemical immunosensor using triple signal amplification strategy for fast silver deposition and electrochemical oxidation of the deposited silver [160]. Copyright 2019 American Chemical Society. (**C**) Schematic illustration of a sandwich-type EC and EN redox-cycling-based electrochemical immunosensor for OMV detection [162]. Copyright 2019 American Chemical Society. (**D**) Schematic illustration of a sandwich-type electrochemical immunosensor for TSH detection based on EC and EN redox cycling [163]. Copyright 2019 American Chemical Society.

DT-D serving as a redox enzyme can catalyze the conversion of an electrochemically inactive substrate with a nitro group into an electroactive product with an amine group [158]. Kang et al. developed an electrochemical immunosensor for the detection of PTH using DT-D as the bifunctional enzyme label for enzymatic amplification and redox cycling [159]. As displayed in Figure 14A, to minimize the direct reaction between nitro(so) compounds with NAD(P)H, six compounds containing a nitro or nitroso group were tested in terms of signal-to-background ratio. As a result, 4-nitroso-1-naphthol (4-NO-1-N) was selected as the enzyme substrate used to develop a DT-D-based sandwich-type immunosensor. DT-D catalyzed the reduction of 4-NO-1-N to 4-NH_2_-1-N by NADH (reaction i). The generated 4-NH_2_-1-N was electrochemically oxidized at the avidin-modified ITO electrode (reaction ii). The oxidized form of 4-NH_2_-1-N could be directly reduced back to 4-NH_2_-1-N by NADH and electrochemically oxidized again (reaction iii), which corresponded to EC redox cycling. Meanwhile, the oxidized species could be regenerated by NADH with the aid of DT-D (reaction iv), corresponding to EN redox cycling. Consequently, the combination of enzymatic catalysis and EC as well as EN redox cycling produced a highly amplified electrochemical signal. The electrochemical immunosensor achieved a wide linear range and a low detection limit (2 pg/mL). However, the DT-D-catalyzed soluble signaling species may diffuse away from the electrode surface during the incubation period, leading to a decreased electrochemical signal. To address this problem, Bhatia et al. reported an ultrasensitive method for the detection of PTH by combining the DT-D-catalyzed nitroso reduction and redox cycling with fast silver deposition (Figure 14B) [160]. In this work, the DT-D-based enzymatic generation of reductive 4-NH_2_-1-N catalyzed the reduction of Ag^+^ to insoluble Ag deposition on the ITO electrode. The oxidized form of 4-NH_2_-1-N was reduced back into 4-NH_2_-1-N by NADH through the CC (ii + iii) and CN (ii + iv) redox cycling process. Under the triple signal amplification (enzymatic amplification and CC and CN redox cycling), the generated silver deposition on the ITO electrode surface was electrochemically oxidized to produce a strong signal. By virtue of EE redox cycling, this electrochemical immunosensor showed a wide range from 100 fg/mL to 100 ng/mL and a detection limit of ~100 pg/mL, which was lower than that of the immunosensor using 4-NH_2_-1-N as the soluble signal species (2 pg/mL) [159]. However, this method required a washing step after Ag deposition because the DT-D substrate can be oxidized during the electrochemical oxidation of Ag deposition. To simplify the electrochemical assays and avoid the oxidation of the DT-D product, Bhatia et al. proposed a simple and fast method for Ag deposition using DT-D as the enzyme label and CN redox cycling of 1,4-naphthoquinone (NQ) by NADH [161].

Ichzan et al. found that DT-D could catalyze the reductive dephosphorylation of a phosphate-containing substrate using NADH or NADPH as the reductant [162]. Then, they developed a sandwich-type electrochemical immunosensor for the detection of Gram-negative bacterial outer membrane vesicles (OMVs). As illustrated in Figure 14C, 1-amino-2-naphthyl phosphate (ANP) exhibited the highest electrochemical signal-to-background ratio compared with 4-aminophenyl phosphate and AAP. The EC and EN redox cycling of dephosphorylated product 1-amino-2-naphthol (AN) by NADH at low electrocatalytic ITO electrodes could generate a highly amplified electrochemical signal. Meanwhile, Nandhakumar et al. demonstrated that DT-D from *Bacillus stearothermophilus* possessed high carboxyl esterase-like activity in the presence of NADH and developed an electrochemical immunosensor for TSH detection using DT-D as the enzyme label [163]. The detailed working principle is shown in Figure 14D. The products generated from DT-D enzymatic catalysis participated in the fast EC and EE redox cycling by NADH, realizing the triple amplification and sensitive detection of TSH.

**Figure 15:**
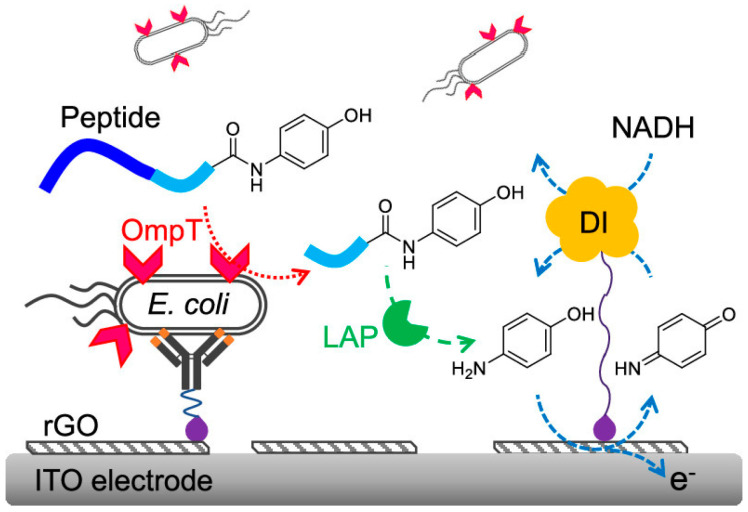
Schematic illustration of wash-free amperometric *E. coli* detection based on the sequential proteolytic cleavage by OmpT and LAP, and EN redox cycling [168]. Copyright 2022 American Chemical Society.

In addition, DT-D can participate in EN redox cycling with other enzymes, such as ALP [164], β-galactosidase [57], lactate dehydrogenase [165], and nicotinamide adenine dinucleotide (NAD)-dependent NAD-GDH [166]. Campàs et al. constructed a competitive electrochemical immunosensor for the detection of okadaic acid (OA) using ALP as the enzyme label and EN redox cycling of *p*-AP by DT-D and NADH [167]. Park et al. reported a wash-free amperometric detection of *E. coli* based on DT-D EN redox cycling (Figure 15) [168]. In this study, *E. coli* was captured by the anti-*E. coli* IgG-modified ITO electrode. The cell membrane endopeptidase of *E. coli*, OmpT could cleave the peptide bond in the substrate containing the segment of alanine–arginine–arginine–leucine–AP (A-R-R-L-AP). The generated R-L-AP was further hydrolyzed by leucine aminopeptidase (LAP), releasing an electroactive species AP that could trigger the EN and EC redox cycling in the presence of DT-D and NADH. Based on the two-sequential enzymatic cleavage and EN redox cycling, *E. coli* in tap water was determined with a detection limit of 10^3^ CFU/mL.

### 3.7. Nanocatalysts or Artificial Enzymes

**Figure 16:**
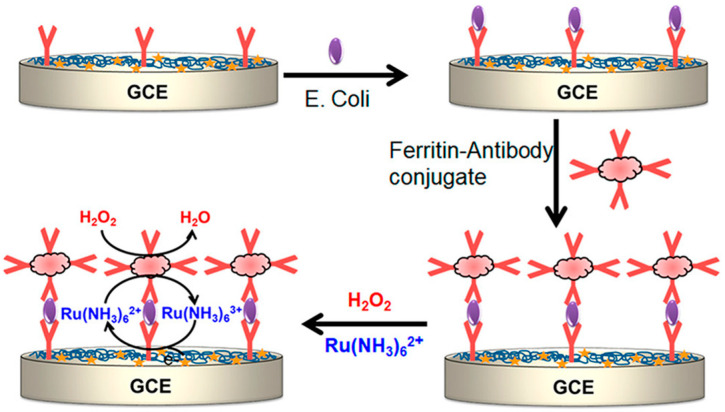
Schematic illustration of the preparation of electrochemical immunosensor and immunoassay procedure with ferritin-triggered redox cycling [178]. Copyright 2018 American Chemical Society.

Despite the wide applications of natural enzymes, they still have several drawbacks, including poor environmental stability, high cost, difficulty in storage, and strict working conditions. To address these limitations, artificial enzymes have been exploited to mimic natural enzymes more effectively, including organic molecules, organic complexes, DNAzymes, and nanomaterials with enzyme-like characteristics (i.e., nanozymes) [169,170,171]. These artificial enzymes have been widely used to construct electrochemical biosensors for the quantitative detection of disease biomarkers [172,173,174]. For instance, ferritin containing a ferric nanocore in the hollow protein cage can endow it with HRP-mimic activity [175]. It has been used to develop sandwich immunoassays due to its catalytic activity toward the oxidization of substrates in the presence of H_2_O_2_ [176,177]. Akanda reported an electrochemical immunosensor for the detection of *Enteropathogenic coli* (*E. coli*) antigens using ferritin as a label to trigger electrochemical nanocatalyst redox cycling [178]. As displayed in Figure 16, in the presence of H_2_O_2_, ferritin was oxidized into the oxidized form, which could oxidize Ru(NH_3_)_6_^2+^. The re-generated reduced form of ferritin could catalyze the decomposition of H_2_O_2_ again and the produced Ru(NH_3_)_6_^3+^ was then electrochemically reduced back into Ru(NH_3_)_6_^2+^. Ferritin-based redox cycling resulted in a high signal amplification efficiency and a low background signal.

**Figure 17:**
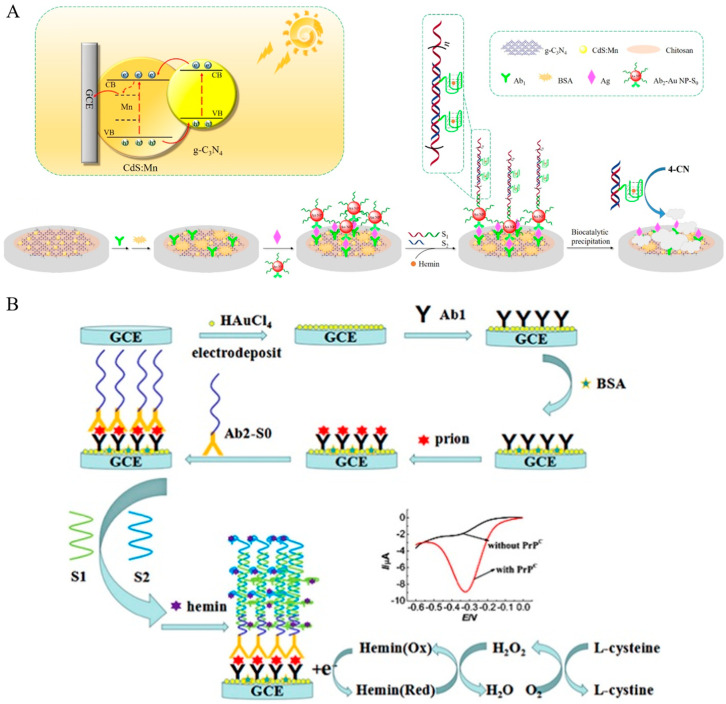
(**A**) Schematic illustration of the mechanism of the photocurrent generation of g-C_3_N_4_/CdS:Mn under visible-light irradiation and schematic illustration of PEC immunosensing system [189]. Copyright 2018 Elsevier. (**B**) Schematic illustration of the electrochemical immunosensor based on HCR and hemin/G-quadruplex DNAzyme for signal amplification [192]. Copyright 2018 Elsevier.

Guanine-rich nucleic acid sequences can form G-quadruplex structures in the presence of cations (e.g., K^+^, Pb^2+^, and NH^4+^) and further bind with hemin to form hemin/G-quadruplex HRP-mimicking DNAzymes. Such artificial enzymes can be used for sensing events by catalyzing H_2_O_2_-mediated oxidation [179,180,181]. Although hemin in the DNAzyme can be directly measured by voltammetric techniques [182,183,184], EC redox cycling between hemin and additional oxidants can amplify the cathodic current [185,186]. Therefore, hemin/G-quadruplex-based DNAzymes have been widely used as electrocatalysts and biolabels for electrochemical immunoassays. For example, Tang et al. developed a sandwich-type electrochemical immunosensor for human IgG1 detection based on DNAzyme-containing DNA concatemers, which were formed by the self-assembly of short DNA fragments via a hybridization chain reaction (HCR). The dendritic DNA strands with rich G bases could bind with hemin to form hemin/G-quadruplexes, termed as DNAzyme concatemers [187]. In addition, electron mediators can enhance the efficiency of the electron transfer between the hemin and electrode and avoid the influence of dissolved oxygen, improving the detection sensitivity [188]. Zhang et al. reported the photoelectrochemical immunoassay of PSA by coupling DNAzyme concatemers with enzymatic biocatalytic precipitation [189]. As illustrated in Figure 17A, CdS:Mn/g-C_3_N_4_ nanohybrids were employed as photoactive materials and modified with capture antibodies. AuNPs was used to load the initiator strand and detection antibody. After the immune-reaction and HCR reaction, many DNAzymes formed between DNA concatemers and hemin effectively catalyzed the precipitation reaction toward 4-chloro-1-naphthol, thus leading to a decrease in the photocurrent. In addition, hemin/G-quadruplex DNAzymes can also be used as biocatalysts for driving other biocatalytic transformations under aerobic conditions, including the NADH oxidase-like oxidation of NADH to NAD^+^ and the oxidation of thiols to disulfides [190,191]. In this view, Li et al. reported the electrochemical immunoassay of prion proteins by integrating HCR with hemin/G-quadruplex DNAzymes for signal amplification (Figure 17B) [192]. In this study, the formed hemin/G-quadruplex DNAzyme could catalyze the aerobic oxidation of L-cysteine to L-cystine, accompanied by the generation of H_2_O_2_. The hemin/G-quadruplex was oxidized by H_2_O_2_ and then immediately electrochemically reduced back into the reduced formation at the electrode surface. The redox cycling of hemin in the presence of L-cysteine and dissolved oxygen resulted in an increase in the reduction current.

**Figure 18:**
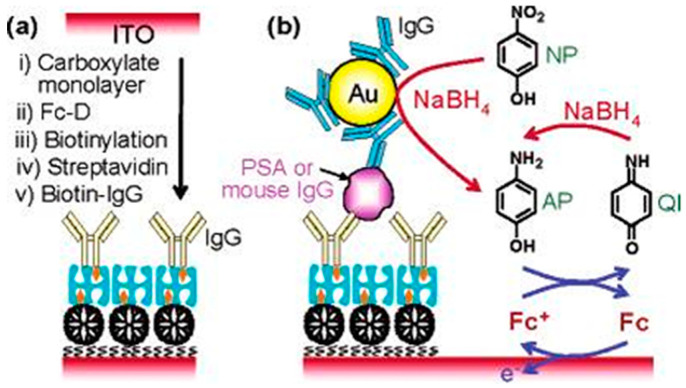
Schematic representation of the preparation of an immunosensing layer (**a**). Schematic view of electrochemical detection of mouse IgG or PSA (**b**). Reprinted with permission from reference [200]. Copyright 2018 American Chemical Society.

**Figure 19:**
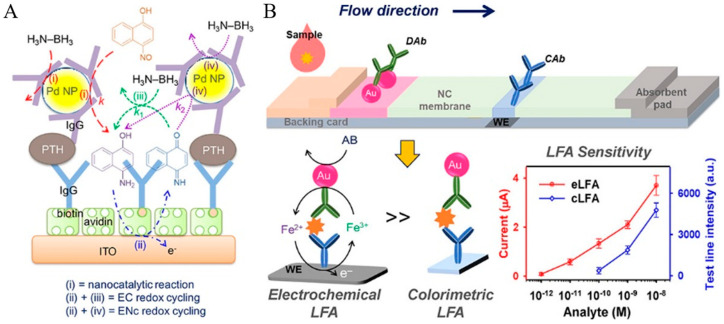
(**A**) Schematic illustration of an electrochemical immunosensor using Pd NPs as nanocatalytic labels [203]. Copyright 2018 American Chemical Society. (**B**) Schematic illustration of the immuno-reaction scheme using Fe^3+^/AuNP/AB and TMB/HRP/H_2_O, in which Fe^3+^ and Fe^2+^ represent [Fe(CN)_6_]^3−^ and [Fe(CN)_6_]^4−^, respectively [204]. Copyright 2023 American Chemical Society.

Nanoparticles with catalytic or enzyme-mimic ability (nanozymes) have received wide attention as catalytic labels for the signal-amplified detection of biorecognition events [193,194]. The nanocatalytic reactions can be integrated with redox cycling systems [195,196,197,198,199]. For example, Das et al. reported an electrochemical immunosensor for PSA detection based on gold nanoparticles (AuNPs) as nitrosoreductase-like nanocatalysts and ECC redox cycling amplification [200]. As displayed in Figure 18, a partially ferrocenyl-tethered dendrimer (Fc-D) deposited on an ITO electrode was sequentially modified with biotin, streptavidin, and biotin-labeled IgG. After the formation of a sandwich immune-complex, AuNPs catalyzed the reduction of *p*-nitrophenol (NP) into *p*-aminophenol (AP) in the presence of NaBH_4_. AP was immediately electrochemically oxidized into *p*-quinone imine (QI) with Fc as the electron mediator, and QI was reduced back to AP by the additional reducing reagent of NaBH_4_ and then re-oxidized at the electrode. The ECC redox cycling of AP could greatly increase the oxidation current of AP and significantly amplify the detection signal. Meanwhile, the slow electron transfer kinetics of NaBH_4_ on the ITO electrode resulted in a low background signal and the electronic mediation of Fc lowered the oxidation potential of AP. Furthermore, Yang’s group introduced magnetic beads into the immunoassays for mouse IgG detection [201]. Tang et al. reported the detection of α-fetoprotein using carbon-nanotube-enriched AuNPs as the nanolabels/nanocatalysts [202]. However, enzyme-like catalytic reactions always suffer from the problems of a low reaction rate and side reaction in O_2_-dissolved electrolyte solution. In addition, NaBH_4_ may undergo self-hydrolysis to generate many bubbles. To overcome these shortcomings, Nandhakumar et al. reported a redox-cycling-based immunosensor for the detection of PTH using 4-NO-1-N, Pd NPs, and H_3_N−BH_3_ [203]. As presented in Figure 19A, Pd NPs catalyzed the reduction of 4-NO-1-N into 4-NH_2_-1-N with H_3_N−BH_3_ as the reducing agent. 4-NH_2_-1-N was electrochemically oxidized at the ITO electrode and then regenerated through the reduction of H_3_N−BH_3_. Nandhakumar et al. reported a lateral flow immunosensor based on electrochemical nanocatalyst redox cycling using ferro/ferricyanide ([Fe(CN)_6_]^3−/4−^, Fe^3+/2+^), ammonia−borane (H_3_N−BH_3_, AB), and AuNP as the mediator, a reducing agent, and a catalytic label (Figure 19B), respectively [204]. In this work, Fe^3+^ was nanocatalytically reduced to Fe^2+^ in the presence of AuNP and AB, which produced a high current for electrochemical detection. Compared with the standard HRP-based enzymatic redox cycling, the detection platform with Fe^3+^/AuNP/AB-based electrochemical-nanocatalyst redox cycling enables better sensitivity, allowing for the detection of insulin with a detection limit down to 12 pM.

**Figure 20:**
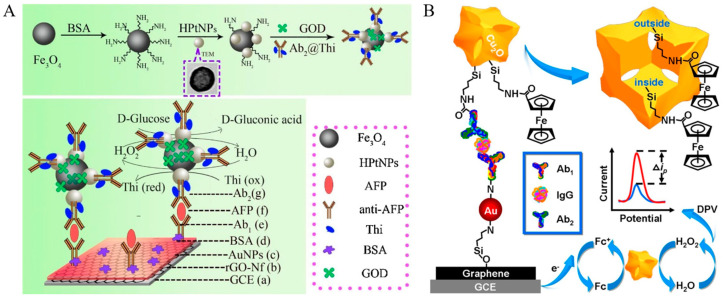
(**A**) Schematic illustration of the preparation of the GOx/HPtNPs-Fe_3_O_4_ and the stepwise immunosensor fabrication process [209]. Copyright 2014 Elsevier. (**B**) Schematic illustration of the sandwich-type electrochemical immunosensor using cubic Cu_2_O nanoframes as the HRP-mimicking label [210]. Copyright 2016 Elsevier.

Thanks to the remarkable achievements in nanotechnology and nanoscience, more and more nanozymes have been introduced as the labels to replace natural enzymes for the development of redox-cycling-based biosensors [205,206]. Compared with natural enzymes, these nanozymes exhibit the merits of high stability, low cost, easy production, and tunable catalytic activity. Among them, Fe_3_O_4_ nanoparticles with enzyme-mimetic activity have been widely applied in the development of electrochemical biosensors [207,208]. For instance, Yang et al. developed a pseudo-bienzyme electrochemical immunosensor for the detection of AFP using hollow platinum-modified Fe_3_O_4_ nanoparticles (HPtNPs-Fe_3_O_4_) as the peroxidase mimetic and GOx (Figure 20A) [209]. In this study, both HPtNPs and Fe_3_O_4_ nanoparticles show peroxidase-like catalytic ability to catalyze the oxidation of thionine by H_2_O_2_ that was produced through the GOx-catalyzed oxidation of glucose. Under the HPtNPs-Fe_3_O_4_-based EN_c_ redox cycling of thionine, the reduction current was greatly improved. In addition, Ma et al. employed cubic Cu_2_O nanoframes as the HRP-mimicking labels to design electrochemical immunosensors [210]. As presented in Figure 20B, AuNPs-decorated 3-aminopropyltriethoxysilane-functionalized graphene sheets (Au@APTES-GS) were used to immobilize Ab_1_. Cu_2_O nanoframes were utilized to carry Ab_2_ and the redox mediator (ferrocenecarboxylic acid, Fc-COOH). Cu_2_O nanoframes with HRP-like activity could catalyze the oxidation of Fc-COOH by H_2_O_2_. The oxidized Fc-COOH was electrochemically reduced back into Fc-COOH immediately for the subsequent H_2_O_2_-mediated oxidation. Finally, the EN_c_ redox cycling of Fc-COOH dramatically amplified the electrochemical signal. In addition, carbon-based nanostructures have been demonstrated to possess peroxidase-like catalytic activity, including graphene oxide and graphene quantum dots. Luo et al. used the nanocomposite of single-wall carbon nanotubes and a graphene quantum dots composite to catalyze the reaction between H_2_O_2_ and thionine for the detection of CEA [211].

## 4. Hydrolytic Enzymes as Signal Labels

### 4.1. ALP-Based Redox Cycling

#### 4.1.1. ALP-Based EC Redox Cycling

**Figure 21:**
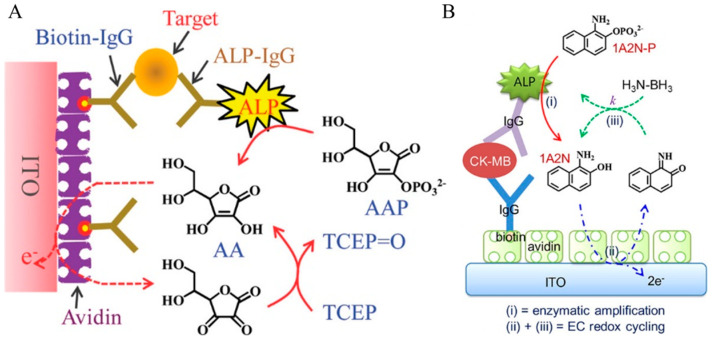
(**A**) Schematic illustration of an electrochemical immunosensor using the generation of AA by ALP and the EC redox cycling of AA by TCEP [220]. Copyright 2011 American Chemical Society. (**B**) Schematic illustration of an electrochemical immunosensor using (i) enzymatic amplification and (ii) + (iii) EC redox cycling [222]. Copyright 2017 American Chemical Society.

ALP can catalyze the hydrolysis of orthophosphoric monoesters into alcohols or phenols. In view of its high turnover frequency and excellent stability, ALP has been widely used as the enzyme label in immunoassays for signal amplification [212,213]. The enzyme can catalyze the hydrolysis of electrochemically inactive substrates such as *p*-aminophenyl phosphate (*p*-APP) and L-ascorbic acid 2-phosphate (AAP) into an electroactive product of *p*-AP and AA, which can be electrochemically oxidized into *p*-quinone imine (QI) and dehydroascorbic acid (DHA), respectively [214,215,216]. To improve the detection sensitivity, reducing agents can be added to regenerate the enzymatic electroactive species after their electrochemical oxidation. In addition, reducing agents can also prevent the oxidation of enzymatic products by O_2_. To minimize the background current, electrochemical immunosensors require the use of ITO electrodes with low electrocatalytic activities for the additional reducing agents. However, ITO electrodes with low electroactivity show a low electrochemical oxidation rate for ALP enzymatic products, leading to a weak signal. Therefore, it is important to select the appropriate ALP enzymatic product, reducing agent, and sensing electrode. In *p*-AP redox cycling, several reducing agents have been used to regenerate *p*-AP, including NaBH_4_, hydrazine, and tris(2-carboxyethyl)phosphine (TCEP) [217,218]. The electro-oxidation of the ALP enzymatic product (*p*-AP) is slow at the ITO electrode and it is necessary to modify the electrode with electron-mediating species. Aiming to achieve a high signal-to-background ratio, ALP substrates should be electrochemically inactive and the corresponding ALP enzymatic products should be electrochemically oxidized at a low formal potential and high reaction rate [219]. Akanda et al. developed an ALP- and AAP-based EC redox-cycling-based immunosensor for the detection of troponin I (Figure 21A) [220]. In this work, the performances of AAP are compared with that of other ALP substrates (e.g., 1-naphthyl phosphate (NPP) and 4-amino-1-naphthyl phosphate (ANP)). The results indicate that AAP and AA are a better substrate and product than others in terms of the formal potential and electro-oxidation rate. Avidin-modified ITO electrodes without the immobilization of an electron mediator exhibited good voltametric behavior regarding the fast electro-oxidation of AA. TCEP showed a fast recycling reaction with low anodic current at the ITO electrode. The EC redox-cycling-based method exhibited a detection limit of 10 fg/mL for the detection of troponin I. In addition, the redox cycling of AA by TCEP could also be used to develop electrochemical immunosensors for the detection of *Salmonella* [221].

Aromatic dihydroxy and aminohydroxy compounds, including monoaromatic and diaromatic compounds, have been widely used as electroactive species for signal output in electrochemical biosensors due to their fast and two-electron redox reactions. Generally, the electrochemical oxidation of diaromatic compounds is faster than that of monoaromatic compounds. However, diaromatic compounds can be rapidly oxidized by dissolved O_2_ and the electrochemical reduction of the oxidized diaromatic compounds may suffer from the interference from O_2_. Although *ortho*-substituted aromatic dihydroxy and aminohydroxy compounds undergo faster electrochemical and catalytic reactions than the para-substituted compounds, they are susceptible to oxidation polymerization by dissolved O_2_ and subsequent nucleophilic addition. To exploit more ALP substrates and reductants for redox cycling, Seo et al. evaluated the performances of four strong reductants and nine aromatic dihydroxy and aminohydroxy compounds for the construction of effective EC redox cycling systems (Figure 21B) [222]. The results demonstrated that the combination of 1-amino-2-naphthol (1A2N) and H_3_N-BH_3_ led to a high signal-to-background ratio. The presence of excess H_3_N-BH_3_ could significantly prevent the oxidation and polymerization of 1A2N by dissolved O_2_. As a proof-of-concept, creatine kinase-MB (CK-MB) was detected in a wide linear range with a low detection limit of 80 fg/mL.

**Figure 22:**
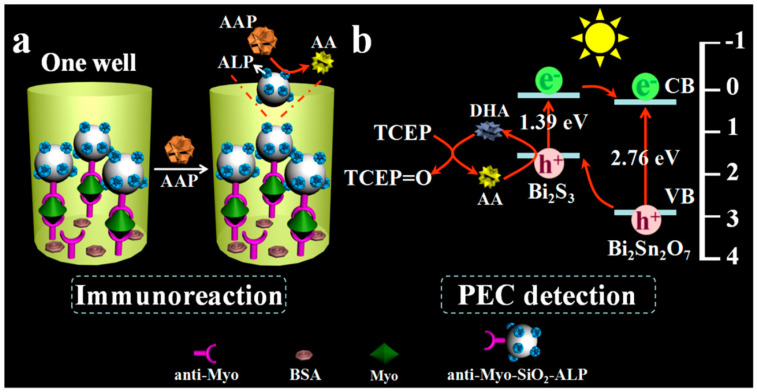
Schematic illustration of (**a**) sandwich immunoreaction in 1 well of 96-well plate and ALP-catalyzed generation of AA, (**b**) redox cycling for signal amplification on Bi_2_S_3_/Bi_2_Sn_2_O_7_ heterojunction [227]. Copyright 2018 American Chemical Society.

In EC redox-cycling-involved electrochemical bioassays, enzymatic products as the signaling species are electrochemically reduced or oxidized at the electrode and then immediately regenerated by additional reducing agents. Similarly, the photogenerated holes at the photoelectrode can also oxidize or reduce enzymatic products to trigger the redox cycling process for signal amplification [223,224,225,226]. In 2018, Cao et al. first combined photogenerated-hole-induced chemical redox cycling with a split-type PEC immunoassay for myoglobin detection [227]. As displayed in Figure 22, ALP catalyzed the generation of AA as an electron donor, which was then oxidized by the photogenerated holes of a Bi_2_S_3_/Bi_2_Sn_2_O_7_ heterojunction photoelectrode. In the redox cycling process, the generated DHA was reduced by the reducing agent TCEP for repeated oxidation at the photoelectrode. The efficient regeneration of the electron donor AA resulted in the enhancement of the photocurrent response. Based on this principle, the developed PEC immunoassay allowed for the detection of myoglobin in a linear range from 4.0 × 10^−13^ to 1.0 × 10^−7^ g/mL. Besides the regeneration of enzymatic product AA by the additional reducing agents, the redox mediators that were oxidized by the photogenerated holes can also be regenerated by the enzymatic products, significantly amplifying the PEC signal [228,229].

**Figure 23:**
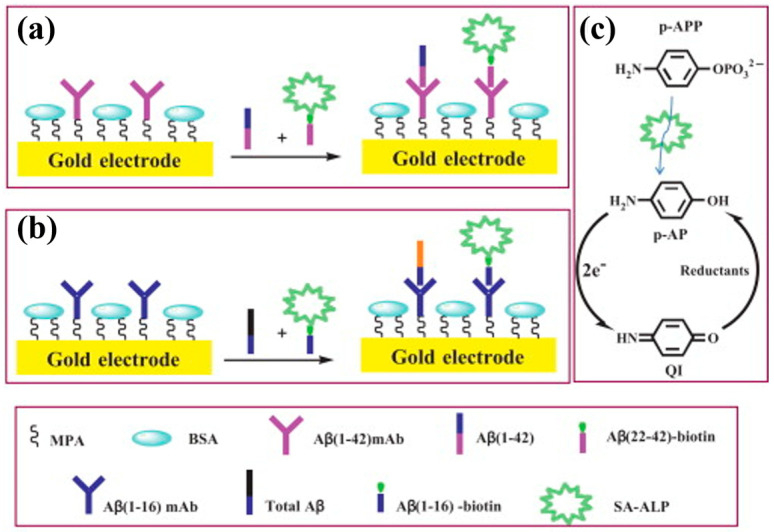
Schematic illustration of the detection of A*β*(1–42) (**a**) and total Aβ (**b**) using *p*-AP redox cycling by chemical reductants (**c**) [234]. Copyright 2014 Elsevier.

Highly electrocatalytic gold electrodes are not suitable for redox cycling by general reducing agents because the redox reaction of them with a low oxidation potential at the gold surface will result in a high background signal. Therefore, there remains a great potential to exploit effective reducing agents for *p*-AP redox cycling with a low background current. Our group systematically evaluated the performances of biosensors in the presence of different reducing agents, including NaBH_4_, hydrazine, TCEP, NADH, NaSO_3_, and cysteamine, on the alkanethiol-covered gold electrodes [230]. The results suggest that the electrocatalytic ability of good electrodes was depressed by the insulating alkanethiol self-assembled monolayers (SAMs) [231,232,233] and that the performances in the case of TCEP and cysteamine were better than those of others. Then, our group developed a competitive electrochemical immunosensor for the detection of *β*-amyloid(1–42) (A*β*(1–42)) and total *β*-amyloid peptides based on *p*-AP redox cycling [234]. As shown in Figure 23, the conjugates of A*β*(22–42)-biotin-SA-ALP and Aβ(1–16)-biotin-SA-ALP were employed to competitively bind with monoclonal antibodies attached on the electrode surface. In the presence of TCEP, the enzymatic product (*p*-AP) was recycled through EC redox cycling, greatly amplifying the electrochemical signal.

#### 4.1.2. ALP-Based ECC Redox Cycling

**Figure 24:**
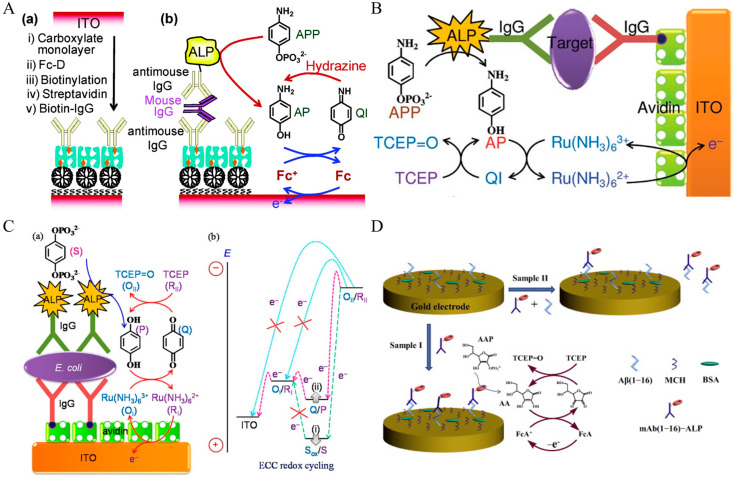
(**A**) Schematic illustration of the preparation of an immunosensing layer (**a**) and the electrochemical detection for mouse IgG based on ECC redox cycling (**b**) [235]. Copyright 2007 American Chemical Society. (**B**) Schematic illustration of ECC redox cycling for ultrasensitive immunosensors [243]. Copyright 2012 American Chemical Society. (**C**) Schematic illustration of (**a**) *E. coli* O157:H7 detection based on the ECC redox cycling that involves HQ (P), which is enzymatically generated from HQDP (S), (**b**) three-step electron transfer (purple lines) and unwanted electron transfer (green and sky-blue lines) in ideal ECC redox cycling [244]. Copyright 2013 American Chemical Society. (**D**) Schematic illustration of A*β* detection by ALP-based signal amplification combined with AA-triggered ECC redox cycling on an SAM-covered gold electrode [245]. Copyright 2014 Elsevier.

In the ECC redox cycling system, electroactive species are usually used as the electron mediators to accelerate the electrochemical oxidation of enzymatic products on the electrode. Meanwhile, the electron mediators can shift the oxidation potential of enzymatic products, avoiding the possible oxidation of the reductants and reducing the background current. In 2006, NaBH_4_ was firstly used as the reducing agent to develop the nanocatalyst-driven ECC redox cycling of *p*-AP [200]. However, the poor stability of NaBH_4_ at neutral or acidic solutions may limit the performance of the NaBH_4_-involved ECC redox cycling system. Thus, it is urgent to choose more stable reducing agents for ECC redox cycling. In 2007, Das et al. presented an electrochemical immunosensor for the detection of mouse IgG based on *p*-AP redox cycling using electrochemically inactive hydrazine as the reducing agent (Figure 24A) [235]. To obtain a low background current, an ITO electrode was used because of its low electrocatalytic activity and weak capacitive current. Meanwhile, Fc-D was immobilized on the ITO electrode to enhance the electron transfer kinetics of *p*-AP in the presence of hydrazine. After the formation of sandwich immune complexes, *p*-APP was converted into *p*-AP, which could be electro-oxidized into *p*-QI at the Fc-D-modified ITO electrode. In the process, *p*-QI was immediately reduced back into *p*-AP by hydrazine, leading to signal amplification. The ECC redox cycling caused an increase in the current and thus improved the sensitivity.

NADH is a good reducing agent that can be utilized in EN redox cycling under the catalysis of diaphorase [236,237,238]. Moreover, a high over-potential is required for the electrochemical oxidation of NADH at noble metal electrodes [239]. In this view, Kwon et al. reported an electrochemical immunosensor for the determination of mouse IgG based on ECC-based *p*-AP redox cycling by NADH [240]. In this study, the gold electrode was modified with an SAM of long thiol molecules to reduce the background current and further modified with Fc-D to facilitate the oxidation of *p*-AP. NADH showing a slow electrochemical oxidation rate at the gold electrode exhibited a fast chemical reaction with *p*-AP for redox cycling.

As a commonly used reducing agent, TCEP can reduce electroactive species such as DHA and quinone (QI) at a fast rate [241,242]. More importantly, TCEP is resistant to oxidation by O_2_ and is very stable in a wide range of pH values. These characteristics are beneficial to the EC and ECC redox cycling systems. Akanda et al. reported an ECC redox cycling system for the ultrasensitive immunoassay of cardiac troponin I (Figure 24B) [243]. In this work, Ru(NH_3_)_6_^3+^, a QI/AP couple, and TCEP were chosen to design an ECC redox cycling system as the oxidant, enzyme substrate/product, and reductant, respectively. The QI/AP couple facilitated a fast redox reaction with both Ru(NH_3_)_6_^3+^ and TCEP. Under the high signal amplification of ECC redox cycling, this method achieved a detection limit of 10 fg/mL for the detection of cardiac troponin I.

APP is prone to autohydrolysis and is unstable during long-term storage, which may cause unwanted redox cycling and a high background [246]. In addition, *p*-AP is light-sensitive and easily oxidized under air, which is unfavorable during the linear accumulation of *p*-AP for the relatively long-time incubation. Therefore, more ALP substrate/product couples should be explored for the combination of enzymatic amplification and redox cycling [247,248]. Akanda et al. evaluated the performances of five ALP substrate/product couples in terms of signal-to-background ratio and then developed an immunosensor for *E. coli* O157:H7 detection based on hydroquinone diphosphate (HQDP)/HQ-based redox cycling [244]. As displayed in Figure 24C, ALP catalyzed the hydrolysis of HQDP into HQ, which could trigger ECC redox cycling with Ru(NH_3_)_6_^3+^ and TCEP as the oxidant and reductant, respectively. HQ exhibited a lower formal potential than *p*-AP and HQDP exhibited a higher formal potential than APP, leading to faster ECC redox cycling. The immunosensor based on an HQDP/HQ couple had a wide detection range from 10^3^ to 10^8^ CFU/mL. The AAP/AA couple is another suitable ALP substrate/product pair for developing redox cycling because of the easy dissolution of AAP and AA in aqueous solutions, the high formal potential of AAP, and the low formal potential of AA [249]. Our group investigated AA-triggered ECC redox cycling with FcA as the redox mediator at an SAM-covered gold electrode [245]. As displayed in Figure 24D, the ALP-conjugated mAb(1–16) was used to recognize the A*β* peptide. After the competitive immune interaction and the addition of AAP, the production of AA could trigger the ECC redox cycling and generate a strong amperometric signal. As a result, the currents decreased with an increase in A*β* concentration in the range from 1 pM to 0.2 nM with a detection limit of 0.2 pM.

**Figure 25:**
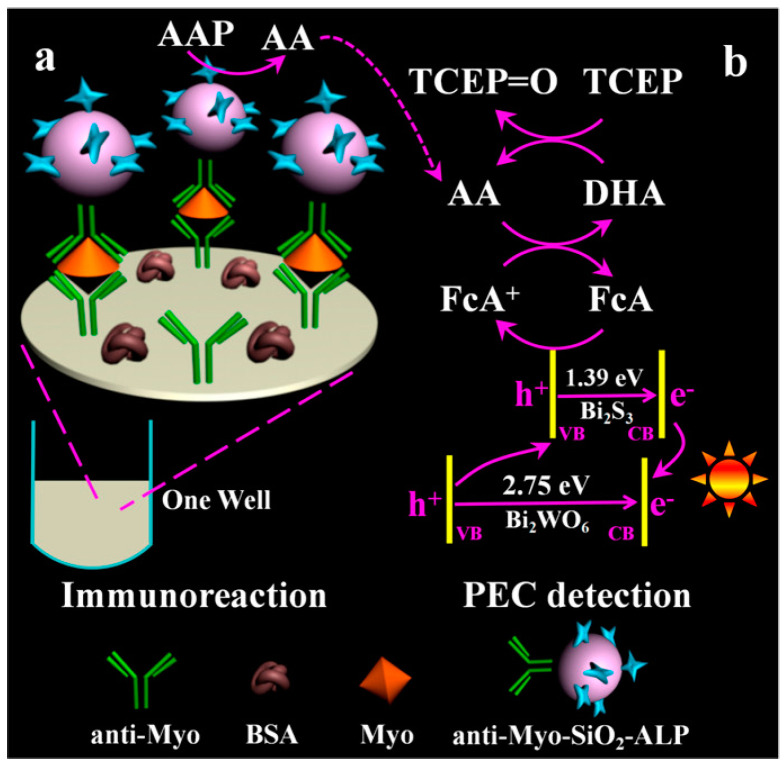
Schematic illustration of (**a**) sandwich immunorecognition and ALP-catalyzed AA formation and (**b**) PECCC redox cycling amplification on Bi_2_S_3_/Bi_2_WO_6_ photoelectrode [256]. Copyright 2018 American Chemical Society.

In redox-cycling-involved electrochemical bioassays, electroactive species are electrochemically reduced or oxidized and then regenerated by other species. In addition, the photogenerated holes at the photoelectrode can also oxidize electroactive species to trigger redox cycling for signal amplification [250,251,252]. Liu’s group developed several photoelectrochemical (PEC) immunosensors by combining redox cycling with enzymatic amplification [253,254,255]. For example, they reported the split-type PEC immunoassay of myoglobin based on photoelectrochemical–chemical–chemical (PECCC) redox cycling [256]. As shown in Figure 25, ALP labels in sandwich immune complexes catalyzed the conversion of AAP into AA, which was then transferred into a detection cell containing FcA and TCEP. The redox mediator of FcA was oxidized by the holes in the Bi_2_S_3_/Bi_2_WO_6_ photoelectrode, initiating the PEC redox cycling process. The resulting oxidized product FcA^+^ was reduced back into FcA by AA and the generated DHA was reduced by TCEP for the next regeneration of FcA.

#### 4.1.3. ALP-Based Ag Biometallization

Silver (Ag) enhancement on metal nanoparticles in the presence of a mild reducing agent is a promising signal amplification strategy for bioassays [257,258,259,260,261,262]. However, the slow reduction of silver ions in the absence of nanoparticle labels will result in a high background signal and poor reproducibility. Enzymatically generated reducing products can reduce silver ions in solution, leading to Ag deposition on the electrode, which is termed as biometallization [263,264]. Electrochemical stripping oxidation of the deposited Ag could provide a high electrochemical signal. The large differences in both formal potential and reaction rates with silver ions between the substrates and the products minimized Ag deposition and significantly decreased the background signal [265]. Aiming to achieve high signal amplification, Haque et al. proposed a CC redox-cycling-based enzymatic Ag biometallization strategy for the sensitive detection of creatine kinase-MB [266]. In this study, the enzyme product AP could reduce Ag^+^ into metallic Ag near the immunosensing surface. NADH was chosen as the strong reducing agent to rapidly reduce the oxidized form of AP (QI). To avoid electroless Ag deposition, an avidin- and BSA-modified ITO electrode was used for Ag deposition. The large Ag nanoparticles (AgNPs) deposited on the electrode provided a high electrochemical stripping signal.

#### 4.1.4. ALP-Based EN Redox Cycling

To amplify the electrochemical signal, it is attractive to couple ALP catalysis with other enzymatic reactions for the regeneration of ALP products to trigger EN redox cycling [267,268]. For example, in ALP/tyrosinase bienzymatic systems, ALP catalyzed the dephosphorylation of phenyl phosphate to produce phenol. The ALP product diphenol was catalytically oxidized by tyrosinase to produce quinine, which could be subsequently reduced at the electrode. The regenerated diphenol was then immediately oxidized into quinone by tyrosinase enzymatic catalysis, realizing signal amplification through phenol recycling [269]. Based on this principle, Piao et al. developed an ALP-labeled electrochemical immunosensor using tyrosinase-modified CNTs [270]. In this work, phenol generated from the ALP-catalyzed hydrolysis of phenyl phosphate was enzymatically converted into electrochemically measurable quinone. The produced catechol was repeatedly oxidized by tyrosinase for the next electrochemical reduction. In a scheme of catechol recycling, human IgG was determined with a detection limit of 0.19 ng/mL.

### 4.2. Protease

**Figure 26:**
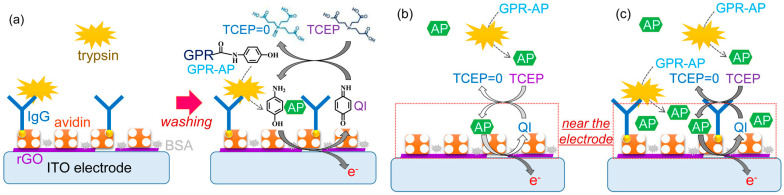
Schematic illustration of three electrochemical trypsin detection methods: (**a**) new trypsin detection using affinity binding, washing process, and proteolytic reaction, (**b**) conventional washing-free trypsin detection using proteolytic reaction, and (**c**) new washing-free trypsin detection using both affinity binding and proteolytic reaction [271]. Copyright 2016 American Chemical Society.

Protease can cleave a peptide bond between two specific amino acid residues. The enzyme plays a vital role in activating or deactivating biological functions in living organisms. To achieve the goal of fast, ultrasensitive, and washing-free detection, Park et al. reported a protease immunosensor based on selective affinity binding, selective proteolytic reaction, and proximity-dependent electrochemical reaction [271]. As illustrated in Figure 26, trypsin was captured by antitrypsin IgG on the electrode surface. The selective proteolytic hydrolysis of an electrochemically inactive *p*-AP-conjugated substrate by trypsin led to the generation of redox-active *p*-AP near the electrode rather than in the bulk solution. The electrochemical oxidation of the released redox-active species near the electrode provided a “signal-on” electrochemical signal, which was amplified by the EC redox cycling reaction. This method exhibited a high signal-to-background ratio and a low detection limit without a washing procedure. Recently, Shin et al. developed an immunosensor for trypsin detection using electrochemical-reduction-based redox cycling [272]. The proteolytically generated *p*-AP was enzymatically or chemically oxidized and then reduced at the electrode. The EC or EN redox cycling of the signaling species by TCEP induced an enhancement in the electrochemical signal.

**Figure 27:**
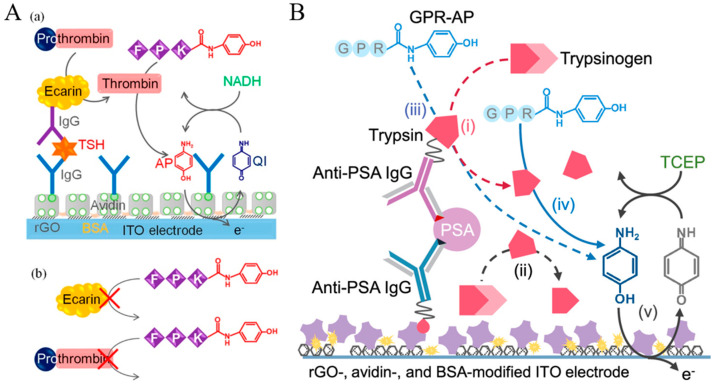
(**A**) Schematic illustration of an immunosensor using a propagating cascade reaction and a redox cycling reaction (**a**), and unwanted proteolytic reactions (**b**) [274]. Copyright 2019 American Chemical Society. (**B**) Schematic illustration of the electrochemical sandwich-type immunosensor based on the autocatalytic activation of trypsinogen by trypsin, the proteolytic cleavage by trypsin, and EC redox cycling [275]. Copyright 2022 American Chemical Society.

The turnover number of proteases is generally lower than that of HRP and ALP, thus limiting their application in immunoassays without additional signal amplification. Two types of proteases can be used to design a propagating cascade reaction with higher signal amplification efficiency than that of a single-proteolytic reaction [273]. Generally, the first enzyme can continually activate the second pro-enzyme and each generated enzyme can produce many signal species. To further lower the detection limit and shorten the incubation period, propagating cascade reactions have been combined with redox cycling. For example, Park et al. reported the electrochemical detection of TSH by coupling the propagating cascade reaction with EC redox cycling (Figure 27A) [274]. In this work, ecarin was employed as the enzyme label to proteolytically transform inactive prothrombin into active thrombin, which could, in turn, cleave the *p*-AP-conjugated peptide substrate. The released electroactive *p*-AP was regenerated through an EC redox cycling reaction in the presence of NADH on the surface of an rGO-modified ITO electrode, leading to a significant increase in the electrochemical signal. In contrast to the propagating cascade reaction using different types of enzymes, the self-propagating autocatalytic reaction can produce signaling species more rapidly. However, the self-activation of the pro-enzyme will result in a high background signal. Recently, Park et al. developed an electrochemical immunosensor for the detection of PSA based on the autocatalytic activation of the trypsinogen mutant by trypsin [275]. As shown in Figure 27B, trypsin was used as the enzyme label for immunoassays and a trypsinogen mutant was selected as the inactive pro-enzyme to minimize the self-activation of trypsinogen. Trypsin generated in a short period of time could catalyze the conversion of the trypsinogen mutant into trypsin, resulting in the release of electroactive *p*-AP from the peptide substrate (GPR-AP). Then, the electrochemical oxidation of AP into QI triggered the EC redox cycling reaction to further enhance the electrochemical signal.

**Figure 28:**
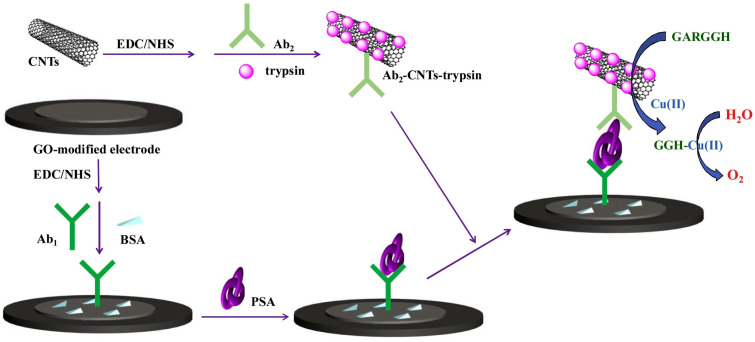
Scheme illustration of the electrochemical immunosensor by the generation of ATCUN-Cu(II) metallopeptides as the electrocatalysts toward water oxidation [278]. Copyright 2019 Elsevier.

Peptides with a specific sequence can bind with metal ions, and the peptide–metal interaction can be modulated by the proteolytic cleavage of the peptide substrate [170]. For example, amino terminal Cu(II)- and Ni(II)-binding (ATCUN) peptides can bind with Cu(II) in a square planar configuration. Such Cu(II)–peptide complexes have been reported to exhibit good electrocatalytic ability toward water oxidation [276,277]. After that, Liu’s group developed an electrochemical immunosensor using trypsin as the signal label for the production of ACTUN-Cu(II) complexes as the electrocatalysts toward water oxidation [278]. As shown in Figure 28, trypsin immobilized on the surface of MWCNTs could cleave the peptide substrates to liberate ATCUN peptides for Cu(II) binding. The Cu(II) center in the formed ACTUN-Cu(II) complex was electrochemically oxidized into Cu(III) and then regenerated by water through an intramolecular coupling mechanism or nucleophilic attack of H_2_O on the high-oxidation-state Cu^IV^=O intermediate. The redox cycling between ACTUN-Cu(II) and H_2_O greatly amplified the electrochemical signal. PSA was determined to be in the linear range of 10 pg/mL–2 ng/mL with a detection limit of 10 pg/mL.

### 4.3. Others

**Figure 29:**
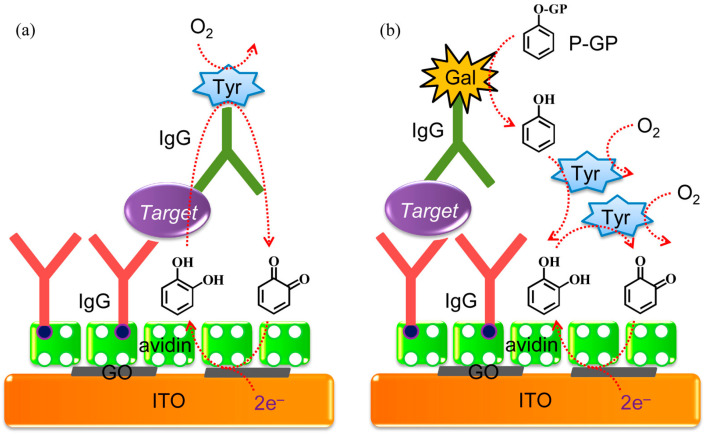
Schematic illustration of (**a**) an electrochemical immunosensor using electro-reduction-based EN redox cycling combined with simultaneous enzymatic amplification (one-enzyme scheme) and (**b**) an electrochemical immunosensor using electro-reduction-based EN redox cycling combined with preceding enzymatic amplification (two-enzyme scheme) [286]. Copyright 2014 American Chemical Society.

*β*-Galactosidase (*β*-Gal) can catalyze the hydrolysis of a *p*-aminophenyl galactopyranoside substrate into electroactive *p*-AP that can be readily determined by EN, EC, and ECC redox cycling [279,280,281]. Therefore, *β*-Gal has become one of the most commonly used labeling enzymes in bioassays [282,283,284,285]. For example, Yang’s group proposed electro-reduction-type EN redox cycling in a two-enzyme scheme for the detection of mouse IgG and CA 15-3 using *β*-Gal and tyrosinase (Figure 29) [286]. Although the ITO electrode showed low electrocatalytic activity toward the electro-reduction of the dissolved O_2_, it exhibited low electrocatalytic activity toward the electro-reduction of *o*-benzoquinone, which was unfavorable in EN redox cycling. Aiming to improve the electrocatalytic activity of the sensing electrode, graphene oxide (GO) was utilized to modify the ITO electrode with a high signal-to-background ratio. *β*-Gal catalyzed the hydrolysis of phenyl *β*-D-galactopyranoside (P-GP) into phenol, which was then oxidized into catechol and *o*-benzoquinone by tyrosinase catalysis. The formed *o*-benzoquinone was electrochemically reduced back into catechol, triggering EN redox cycling at a low potential. The results indicated that the two-enzyme scheme using the GO/ITO electrode showed better performances in terms of signal-to-background ratio, signal intensity, and detection limit.

## 5. Conclusions

In conclusion, we have summarized the recent remarkable progress in the development and application of electrochemical immunosensors by the signal amplification of enzyme- or nanozyme-based catalysis plus redox cycling. Despite the delightful achievements, several challenges in this topic remain to be addressed. First, it is promising to improve the properties of the enzyme label turnover number and substrate affinity (K_m_) and to screen a new immune recognition element with a high binding affinity for the binding target. Second, the environmental factors, including temperature, pH, and activators or inhibitors, may influence the rate of catalytic and redox cycling reactions. More efforts should be devoted to exploring optimized experimental conditions and nanomaterials for the modification of electrodes for effective redox cycling systems. Third, different nanocarriers have been employed to effectively load enzymes for improved sensitivity, but most of the immobilization techniques show the defects of enzyme leaching, denaturation, and limited transfer efficiency. Fourth, despite the advantages of a low cost and high stability, the catalytic activity and efficiency of nanozymes are still lower than those of natural enzymes. Synthesis from different batches and the bioconjugation of nanozymes may result in poor reproducibility. It is a promising approach to expand the types of nanozymes with effective catalytic activity and high specificity for the design of nanocatalyst-based redox cycling.

## Data Availability

Not applicable.
